# Effect of Lifestyle Coaching or Enhanced Pharmacotherapy on Blood Pressure Control Among Black Adults With Persistent Uncontrolled Hypertension: A Cluster Randomized Clinical Trial

**DOI:** 10.1001/jamanetworkopen.2022.12397

**Published:** 2022-05-18

**Authors:** Mai N. Nguyen-Huynh, Joseph D. Young, Bruce Ovbiagele, Janet G. Alexander, Stacey Alexeeff, Catherine Lee, Noelle Blick, Bette J. Caan, Alan S. Go, Stephen Sidney

**Affiliations:** 1 Department of Neurology, Kaiser Permanente Walnut Creek Medical Center, Walnut Creek, California; 2 Division of Research, Kaiser Permanente Northern California, Oakland; 3 Department of Medicine, Kaiser Permanente Oakland Medical Center, Oakland, California; 4 Department of Neurology, University of California, San Francisco; 5 Department of Health Systems Science, Kaiser Permanente Bernard J. Tyson School of Medicine, Pasadena, California; 6 Department of Epidemiology, Biostatistics and Medicine, University of California, San Francisco

## Abstract

**Importance:**

Greater difficulty in controlling blood pressure (BP) and adverse lifestyle practices such as higher salt intake or less physical activity may account for some of the differences between BP control rates in Black vs White adults, thereby exposing Black adults to a higher risk of vascular events.

**Objective:**

To determine whether a lifestyle coaching intervention or an enhanced pharmacotherapy protocol is more effective than usual care in improving BP control rates in Black adults treated within an integrated health care delivery system.

**Design, Setting, and Participants:**

Shake, Rattle & Roll, a cluster randomized clinical trial, was conducted from June 5, 2013, to June 11, 2018, in a large integrated health care delivery system. Enrollment was completed during a 12-month period and interventions were implemented for 12 months. Follow-up lasted 48 months after enrollment. Panels of Black adult members of the health care delivery system with BP of at least 140/90 mm Hg from 98 adult primary care physicians were randomly assigned at the primary care physician level to usual care (UC group [n = 1129]), enhanced pharmacotherapy monitoring (EP group [n = 346]) of current BP management protocol, or diet and lifestyle coaching consisting of photographs, stories, and recipes, for example, that are appropriate for Black adults (LC group [n = 286]) focused on the Dietary Approaches to Stop Hypertension (DASH) diet. Data were analyzed from June 1, 2016, to March 25, 2022.

**Interventions:**

The UC group received care per customary protocol. The EP group was contacted by a research nurse and/or a clinical pharmacist to discuss barriers to hypertension control, and drug therapy emphasized the use of thiazide diuretic intensification and addition of spironolactone as needed. The LC group received as many as 16 telephone sessions with a lifestyle coach and an emphasis on implementing reduction of sodium intake and the DASH diet.

**Main Outcomes and Measures:**

Intention-to-treat analysis of BP control rates at end of the 12-month intervention.

**Results:**

Among the 1761 participants, the mean (SD) age was 61 (13) years, and 1214 (68.9%) were women. At the end of the 12-month intervention period, there was no significant difference in BP control rate among study groups (UC, 61.8% [95% CI, 58.8%-64.9%]; EP, 64.5% [95% CI, 59.0%-69.4%]; LC, 67.8% [95% CI, 62.1%-73.2%]; LC vs EP, *P* = .07). However, greater BP control was present in the LC group vs UC at 24 months (UC, 61.2% [95% CI, 57.3%-64.7%]; EP, 67.6% [95% CI, 61.9%-72.8%]; LC, 72.4% [95% CI, 66.9%-78.1%]; LC vs UC, *P* = .001), and 48 months (UC, 64.5% [95% CI, 61.6%-67.2%]; EP, 66.5% [95% CI, 61.3%-71.3%]; LC, 73.1% [95% CI, 67.6%-77.9%]; LC vs UC, *P* = .006) after enrollment. The contribution of BP medication adherence to explain group differences was inconclusive.

**Conclusions and Relevance:**

In this cluster randomized clinical trial including Black adults with persistent uncontrolled hypertension, a 12-month LC intervention was more effective at controlling BP than UC at 24 and 48 months after enrollment. Further research is needed to explore the potential implementation of this intervention into clinical practice.

**Trial Registration:**

ClinicalTrials.gov Identifier: NCT01892592

## Key Points

**Question:**

Is a 12-month lifestyle coaching intervention or an enhanced pharmacotherapy protocol more effective than usual care in improving blood pressure control in Black adults treated within an integrated health care delivery system?

**Findings:**

In this cluster randomized clinical trial involving 1761 Black adults, the group receiving lifestyle coaching achieved better blood pressure control compared with usual care at 24 and 48 months after enrollment, whereas the group receiving enhanced pharmacotherapy did not.

**Meaning:**

A telephone-based lifestyle intervention focused on the Dietary Approaches to Stop Hypertension diet may help Black adults with uncontrolled hypertension improve blood pressure control in the long term.

## Teaser

This cluster randomized clinical trial evaluates the effectiveness of a lifestyle coaching intervention vs an enhanced pharmacotherapy protocol compared with usual care in controlling blood pressure in Black adults with persistent uncontrolled hypertension.

## Introduction

Black adults have a heavier burden of cardiovascular disease (the leading cause of death) and stroke (the leading cause of long-term disability) in the US compared with White adults.^1,2,3,4,5^ The prevalence of hypertension, the most modifiable risk factor for cardiovascular disease and stroke, is substantially higher in Black adults, who are less likely to achieve adequate blood pressure (BP) control and experience a greater impact on stroke risk compared with White adults.^6,7,8,9,10,11^ Eliminating disparity in BP control could avert thousands of deaths due to cardiovascular disease^12^ and reduce the population burden of dementia.^13^

Rates of hypertension control have improved significantly among members within the Kaiser Permanente Northern California (KPNC) health care delivery system since the establishment of its Preventing Heart Attacks and Stroke Everyday program in 2004.^14,15^ However, a prior cohort study^16^ examining BP control at 6 months after ischemic stroke from KPNC found that Black patients still had poorer BP control compared with patients of other races and ethnicities despite equal use of health care resources, antihypertensive prescriptions, and medication adherence. Greater difficulty in controlling BP and adverse lifestyle practices (eg, higher salt intake, less physical activity, more smoking) may explain this disparity, at least in part.^17,18^ To improve the disparity of BP control between Black and White patients, a population-based approach is necessary for aggressive targeting of high-risk Black adults with uncontrolled BP.^19^ To address the important challenge of treating hypertension in Black adults as emphasized by the International Society on Hypertension in Blacks, our primary aim was to determine whether a lifestyle coaching intervention (LC group) or an enhanced pharmacotherapy monitoring protocol (EP group) would be more effective than usual care (UC group) in controlling BP in Black adults who had persistent uncontrolled hypertension.

## Methods

### Participants and Enrollment

The Shake, Rattle & Roll study was designed to *shake* participants’ salt habit, *rattle* the intensity of KPNC’s pharmacotherapy protocol, and *roll* out the study results. This study was a 3-group, cluster randomized clinical trial with a pragmatic design to test the effectiveness of the intervention in a more generalizable setting. The study was conducted from June 5, 2013, to June 11, 2018, at the Kaiser Permanente Oakland Medical Center, 1 of 21 medical centers within KPNC’s integrated health care delivery system. Primary care physicians (PCPs) with at least 10 or more Black adults in their practice were randomized to 1 of the 3 groups: UC, EP, and LC. Recruitment occurred for 12 months, with interventions delivered for 12 months after enrollment, and the study ended as planned at the end of the funding period. Data collection continued for 36 months after the end of the intervention with no participant contact. The study was approved by the KPNC Institutional Review Board, and patients in the LC and EP groups provided verbal informed consent. Consent was not required for participants randomized to the UC group. This study followed the Consolidated Standards of Reporting Trials (CONSORT) reporting guideline. The study rationale and design have been described in detail elsewhere.^20^ The trial protocol is available in Supplement 1.

Previous research supported that health beliefs could have a significant and lasting impact on preventive health behaviors.^21^ Our interventions were designed with the Health Belief Model^22,23^ in mind to help each participant (1) understand that a bad outcome can be avoided, (2) believe that the bad outcome can be avoided by following the recommended intervention, and (3) believe that he or she can perform the intervention. By increasing a participant’s level of awareness, it would be possible to achieve a higher rate of adherence to the interventions and lead to better BP control.

Electronic medical records (EMRs) in conjunction with the KPNC hypertension registry were used to identify eligible participants. The registry was developed in 2001 and a description has been previously published.^14^ At the time of study initiation, updated clinical practice guidelines on BP^5^ were not in effect, and hypertension was defined as BP of 140/90 mm Hg or higher.

Eligibility criteria included (1) KPNC member with continuous pharmacy benefits during enrollment, (2) 18 years or older at entry, (3) listed in the hypertension registry, (4) self-reported Black or African American race, and (5) sufficient understanding of the English language. Patients who were pregnant, who were receiving home hospice, or who had a documented life expectancy of less than 6 months in their EMR were excluded. After the start of enrollment, exclusion criteria were modified to exclude those with diagnosed dementia owing to concerns of inability to comply with all study activities.

Initial sample size calculations estimated that to have 80% power to detect a difference of 8% (comparing an intervention group and a control group) in the proportion of patients with BP control after 12 months, a sample size of 492 participants in each intervention group and 985 in the UC group was required (2-sided test, α = .05).^20^ Stratified cluster randomization at the level of the PCP was used to assign patients to the LC group (31 PCPs; 286 patients), EP group (34 PCPs; 346 participants), or UC group (33 PCPs; 1129 participants) group (Figure). Strata were defined by the number of eligible patients per PCP patient panel.

**Figure.**
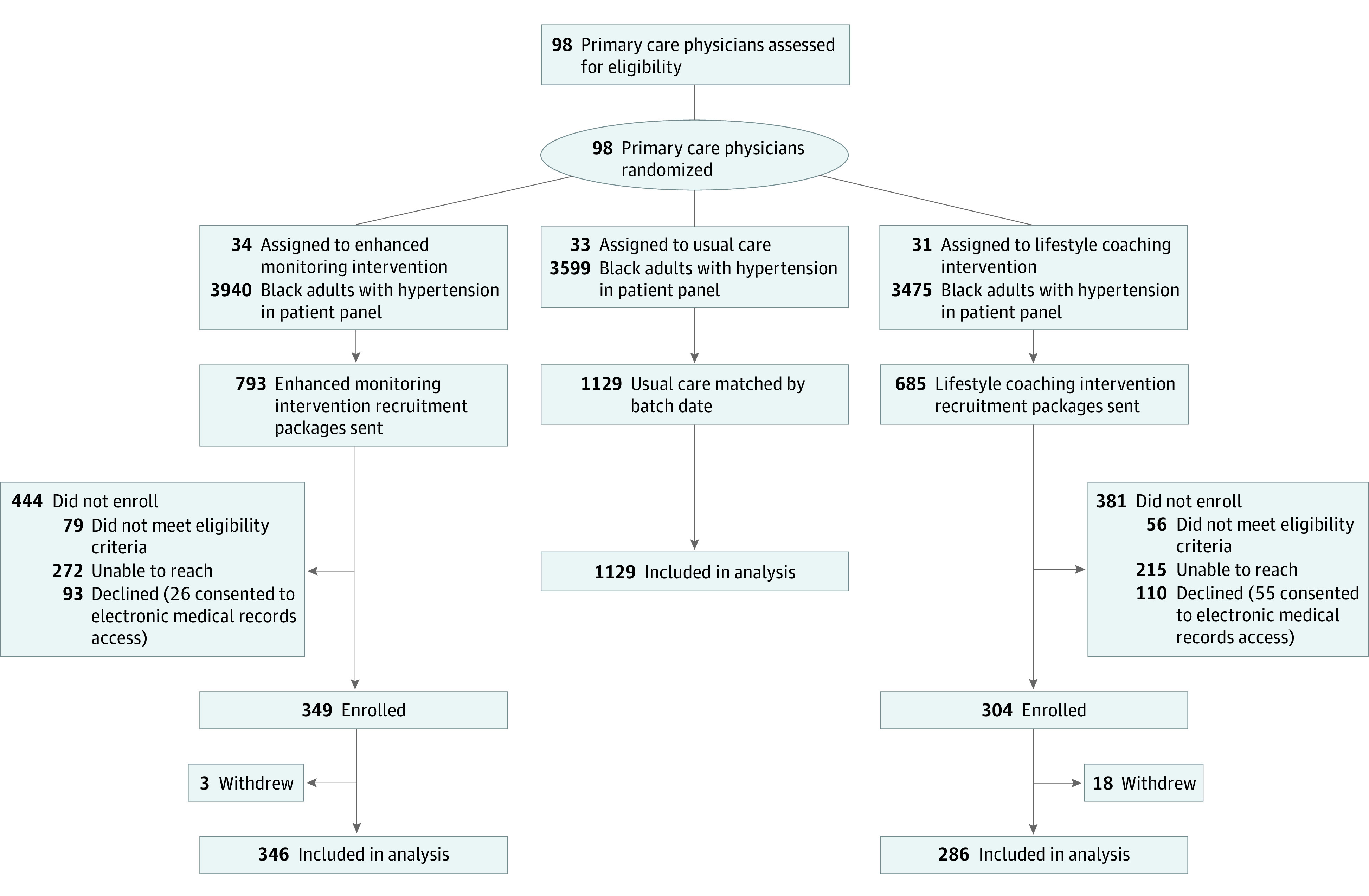
Study Flow Diagram

Participants were enrolled on a rolling basis during a 12-month inception period. Participants left and reentered the eligibility pool depending on whether their BP became controlled or uncontrolled (≥140/90 mm Hg) during the enrollment period. Study staff reached out to obtain verbal consent from participants randomized to the treatment groups in concordance with institutional review board requirements. During each round of enrollment, a proportional number of participants assigned to UC were randomly selected to be included in the study. No harm was observed with any participant during the trial.

### Intervention and UC Groups

Detailed descriptions of each study group have been published previously.^20^ Additional information is available in the eMethods in Supplement 2. Briefly, UC for management of a recent uncontrolled BP measurement in KPNC included free subsequent BP check visits, during which a medical assistant measured and recorded the patient’s BP using an automated BP cuff and reviewed their current BP medications. Findings were reviewed by the PCP or a local pharmacist, and any necessary adjustments to the BP medication regimen were made. In addition, all KPNC members had access to KPNC health education services.

The EP group was based on a prior published KPNC protocol on hypertension management.^14^ This intervention, delivered by a research nurse coordinator and a pharmacist, was designed to expand on UC and increase use of medical assistant BP check visits, to optimize thiazide diuretic dosing, and to increase prescribing of spironolactone for resistant hypertension (ie, receiving ≥2 antihypertensives). The research nurse also raised awareness through education on hypertension and the importance of BP control to reduce the risk of cardiovascular disease and stroke, identified potential barriers to controlling BP, and provided resources within KPNC as appropriate.

The LC group offered participants as many as 16 individual telephone coaching sessions with a lifestyle coach, and the study target was to have at least 50% of the participants completing 6 sessions or more. Both lifestyle coaches were registered dieticians. Motivational interviewing techniques were used to help the participant establish short-term goals related to lifestyle behaviors and to discuss possible barriers to achieving these goals. The main lifestyle goal was to achieve and maintain a low-salt diet based on the Dietary Approaches to Stop Hypertension (DASH) diet.^24,25^ Other session topics included hypertension and its associated cardiovascular risks and addressing barriers to current treatment plans. The emphasis of the coaching sessions was to provide participants with adequate education and resources to be able to conduct recommended lifestyle behaviors on their own at the end of the intervention. Participants had the opportunity to provide informal feedback to study staff before and after the intervention during in-person group meetings.

### Follow-up and Outcome Measures

The primary outcome was the proportion of participants with controlled BP (<140/90 mm Hg) at the end of the 12-month intervention. Because of the pragmatic design, we used BP measurements recorded in the EMR during outpatient visits. Therefore, the timing of measurements varied by patient, and the measurement closest to 12 months (within 3 months before or after) after enrollment was selected. If a participant had 2 BP readings equidistant from the 12-month postenrollment date, then the earlier BP reading was used. Secondary outcomes included the proportion of participants with controlled BP at 24 and 48 months after enrollment. The measurements recorded closest to each point within 3 months before or after were selected for analyses.

For participants who did not have a BP reading within 3 months of a given follow-up point, the last available BP reading after enrollment was carried forward and used in the outcome analysis. If a participant did not have any BP measurement after enrollment (ie, incomplete follow-up data), it was assumed that their BP was uncontrolled at each follow-up point, because all participants had uncontrolled BP at enrollment to qualify for the study.

Sustained BP control was defined as having achieved BP control both at 12 and 24 months after enrollment. No changes to trial outcomes were made after enrollment began.

### Statistical Analysis

Data were analyzed from June 1, 2016, to March 25, 2022. Generalized linear mixed-effect models with a logit link were used to model separately the effect of each intervention on the outcome of BP control (yes or no) at 12, 24, and 48 months after enrollment. Control of BP was dichotomized to mirror the National Committee for Quality Assurance Healthcare Effectiveness Data and Information Set BP quality metric.^26^ All models included treatment group as the exposure and PCP as a random intercept to account for the correlation by PCP owing to cluster randomization. All participants enrolled in the intervention groups were included in the final analysis, regardless of whether the intervention was received (ie, intention to treat [ITT]). *P* values reported were based on Wald-based *F* test with denominator degrees of freedom estimated using the containment method, with 2-sided *P* < .05 indicating statistical significance.^27^ Outcomes were assessed by age and sex subgroups, and we also examined the impact of excluding participants with incomplete follow-up measurements. In sensitivity analyses, BP control (yes or no) was defined using a measure of mean BP, which is obtained through the area under each individual’s BP trajectory curve over the study (denoted as the area under the curve method).^28^ Blood pressure control was analyzed as a longitudinal outcome using linear mixed models and additionally included years since enrollment and a study group by time interaction term that enabled us to assess the temporal variation in group differences of BP control. The former analysis summarizes longitudinally collected BP measures, thereby simplifying the analyses; the latter uses all BP measurements collected during the study. We also included GLIMMIX models of BP control at 12, 24, and 48 months using imputed data (SAS, version 9.04 [SAS Institute, Inc]) (eMethods in Supplement 2).

## Results

The baseline characteristics of enrolled participants by group are given in Table 1. Among 1761 participants, the mean (SD) age was 61 (13) years; 1214 (68.9%) were women and 547 (31.1%) were men; and the mean (SD) body mass index (calculated as weight in kilograms divided by height in meters squared) was 34.0 (8.6). Data on age, sex, body mass index, smoking status, median household income, or comorbidities between groups were similar, except unknown marital status was more common among participants in the UC group (144 of 1129 [12.7%]) compared with those in the LC or EP group (10 of 286 [3.5%] and 21 of 346 [6.1%], respectively). The mean (SD) qualifying BP was 151.0/85.0 (11.4/11.7) mm Hg across all 3 groups.

**Table 1.** Baseline Characteristics of Participants

Characteristic	Treatment^a^		
	UC group (n = 1129)	EP group (n = 346)	LC group (n = 286)
Age, mean (SD), y	60 (13)	60 (13)	61 (12)
Sex			
Women	783 (69.3)	246 (71.1)	185 (64.7)
Men	346 (30.6)	100 (28.9)	101 (35.3)
Baseline BP, mean (SD), mm Hg			
Systolic	150.5 (11.8)	151.5 (11.1)	149.5 (9.9)
Diastolic	84.5 (11.8)	84.7 (11.9)	84.4 (11.3)
Marital status			
Single	591 (52.3)	202 (58.4)	173 (60.5)
Married	394 (34.9)	123 (35.5)	103 (36.0)
Unknown/other	144 (12.7)	21 (6.1)	10 (3.5)
Median household income, mean (SD), $	55 372 (28 027)	52 723 (27 555)	56 621 (28 621)
Medical history			
Diabetes	365 (32.3)	116 (33.5)	102 (35.7)
Heart failure	115 (10.2)	43 (12.4)	40 (14.0)
Coronary heart disease	83 (7.3)	34 (9.8)	23 (8.0)
Stroke	46 (4.1)	12 (3.5)	13 (4.5)
Current cigarette smoker	166 (14.7)	60 (17)	36 (12.6)
BMI, mean (SD)	33.9 (8.7)	34.4 (8.6)	33.6 (8.3)

Abbreviations: BMI, body mass index (calculated as weight in kilograms divided by height in meters squared); BP, blood pressure; EP, enhanced pharmacotherapy monitoring; LC, lifestyle coaching; UC, usual care.

^a^ Unless otherwise indicated, data are expressed as the number (%) of participants. Percentages have been rounded and may not total 100.

Follow-up rates were high in all study groups with few missing outcome data. Overall, 1658 participants (94.1%) had at least 1 BP reading by 12 months after enrollment, 1706 (96.9%) had a BP reading by 24 months after enrollment, and 1724 (97.9%) had a BP reading by 48 months after enrollment. Blood pressure measurements were missing at 12 months for 72 (6.4%) in the UC group, 18 (5.2%) in the EP group, and 13 (4.5%) in the LC group; at 24 months, for 39 (3.5%) in the UC group, 10 (2.9%) in the EP group, and 6 (2.1%) in the LC group; and at 48 months, for 28 (2.5%) in the UC group, 6 (1.7%) in the EP group, and 3 (1.0%) in the LC group. We assumed BP remained uncontrolled for these participants with missing measurements. The distribution of mean BP at baseline and 12, 24, and 48 months after enrollment is presented in eTable 1 in Supplement 2. Examination of the timing of the last BP carried forward at the 3 outcome periods had similar findings across the 3 groups (eTable 2 in Supplement 2).

We performed an ITT analysis that included all enrolled participants (Table 2). At 12 months after enrollment, 698 participants in the UC group (61.8% [95% CI, 58.8%-64.9%]), 223 in the EP group (64.5% [95% CI, 59.0%-69.4%]; *P* = .44 vs UC), and 194 in the LC group (67.8% [95% CI, 62.1%-73.2%]; *P* = .07 vs UC) had controlled BP. At 24 months after enrollment, 691 participants in the UC group (61.2% [95% CI, 57.3%-64.7%]), 234 in the EP group (67.6% [95% CI, 61.9%-72.8%]; *P* = .06 vs UC), and 207 in the LC group (72.4% [95% CI, 66.9%-78.1%]; *P* = .001 vs UC) had controlled BP. At 48 months after enrollment, 728 participants in the UC group (64.5% [95% CI, 61.6%-67.2%]), 230 in the EP group (66.5% [95% CI, 61.3%-71.3%]; *P* = .50 vs UC), and 209 in the LC group (73.1% [95% CI, 67.6%-77.9%]; *P* = .006 vs UC) had controlled BP. In the model of BP control at 12 months after enrollment, the proportion of variance explained by the covariance between PCP groups was less than 1% (intraclass correlation coefficient, 0.004).

**Table 2.** BP Outcomes at 12, 24, and 48 Months After Enrollment

BP outcome^a^	UC group (n = 1129)	EP group (n = 346)	LC group (n = 286)	*P* value		
				EP vs UC	LC vs UC	EP vs LC
**12 mo **						
Participants with BP control, No. (%) [95% CI]	698 (61.8) [58.8-64.9]	223 (64.5) [59.0-69.4]	194 (67.8) [62.1-73.2]	.44	.07	.36
Adjusted OR (95% CI)	1 [Reference]	1.11 (0.85-1.44)	1.30 (0.98-1.73)	NA	NA	NA
**24 mo **						
Participants with BP control, No. (%) [95% CI]	691 (61.2) [57.3-64.7]	234 (67.6) [61.9-72.8]	207 (72.4) [66.9-78.1]	.06	.001	.19
Adjusted OR (95% CI)	1 [Reference]	1.33 (0.99-1.79)	1.71 (1.24-2.36)	NA	NA	NA
**48 mo **						
Participants with BP control, No. (%) [95% CI]	728 (64.5) [61.6-67.2]	230 (66.5) [61.3-71.3]	209 (73.1) [67.6-77.9]	.50	.006	.07
Adjusted OR (95% CI)	1 [Reference]	1.09 (0.85-1.41)	1.50 (1.12-2.00)	NA	NA	NA

Abbreviations: BP, blood pressure; EP, enhanced pharmacotherapy monitoring; LC, lifestyle coaching; NA, not applicable; OR, odds ratio; UC, usual care.

^a^ For BP outcomes at the end of the 12-month intervention, we selected 1 BP reading from 0 to 15 months, but closest to 12 months after enrollment; for 24-month postenrollment follow-up, we selected 1 BP reading from 0 to 27 months, but closest to 24 months after enrollment; and for 48-month postenrollment follow-up, we selected 1 BP reading from 0 to 51 months, but closest to 48 months after enrollment. The 95% CIs and ORs account for clustering by physician using generalized linear mixed models. *P* values correspond to a Wald-based *F* test with denominator degrees of freedom estimated using the containment method.

When we included patients who refused to participate in the interventions but allowed us to examine their EMR data in the ITT model, the LC group had better BP control at 12 months after enrollment compared with the UC group (odds ratio [OR], 1.41 [95% CI, 1.09-1.83]; *P* = .01) (eTable 3 in Supplement 2). The ITT models did not change when we further adjusted for PCP panel size (eTable 4 in Supplement 2) or when we limited the data to include only participants with complete BP data (eTable 5 in Supplement 2). When we stratified the ITT model by age category and sex, being older than 65 years (OR, 1.67 [95% CI, 1.06-2.64]) and female (OR, 1.76 [95% CI, 1.22-2.56]) was associated with better BP control in the LC group compared with the UC group at 48 months after enrollment (eTable 6 in Supplement 2). When we explored the more stringent BP cutoff of less than 130/80 mm Hg as the outcome, fewer participants achieved the cutoff and the rates of BP control were similar across the 3 groups (eTable 7 in Supplement 2).

Using multiple imputation as an alternate approach to handle missing follow-up data, we found that the overall conclusions remained the same, although some ORs were attenuated (eTable 8 in Supplement 2). Results of sensitivity analyses (area under the curve and longitudinal models) support the findings from the ITT analysis that the LC group was more likely to achieve BP control at 24 and 48 months (eResults and eTable 9 in Supplement 2). The percentages of controlled BP over time are shown in eFigure 1 in Supplement 2.

Participants in the LC group were considered to have completed the intervention if they participated in at least 6 sessions (eFigure 2 in Supplement 2). Participants who completed the LC intervention (n = 146) were significantly more likely to achieve BP control at 12 months after enrollment compared with those in the UC group (n = 1129) (107 [73.3%] vs 700 [62.0%]; *P* = .01). This was also observed at 24 months after enrollment favoring the LC group (n = 146) compared with the UC group (n = 1129) (117 [80.1%] vs 691 [61.2%]; *P* < .001). Sustained BP control at 24 months after enrollment was observed in 507 participants in the UC group (44.9% [95% CI, 41.5%-48.4%]), 173 in the EP group (50.0% [95% CI, 44.2%-55.6%]; *P* = .14 vs UC), and 166 in the LC group (58.0% [95% CI, 52.0%-64.1%]; *P* < .001 vs UC) (Table 3).

**Table 3.** Participants With Sustained BP Control at 12, 24, and 48 Months After Enrollment

BP outcome^a^	UC group (n = 1129)	EP group (n = 346)	LC group (n = 286)	*P* value		
				EP vs UC	LC vs UC	EP vs LC
Participants with sustained BP control at 12 and 24 mo, No. (%) [95% CI]	507 (44.9) [41.5-48.4]	173 (50.0) [44.2-55.6]	166 (58.0) [52.0-64.1]	.14	.001	.05
Participants with sustained BP control at 12 and 48 mo, No. (%) [95% CI]	502 (44.6) [41.3-48.4]	166 (48.0) [41.8-53.2]	152 (53.2) [47.1-59.4]	.45	.02	.17

Abbreviations: BP, blood pressure; EP, enhanced pharmacotherapy monitoring; LC, lifestyle coaching; UC, usual care.

^a^ For BP outcomes at the end of the 24-month postenrollment follow-up, we selected 1 BP reading from 0 to 27 months, but closest to 24 months after enrollment; and for 48-month postenrollment follow-up, we selected 1 BP reading from 0 to 51 months, but closest to 48 months after enrollment. The 95% CIs account for clustering by physician using generalized linear mixed models. *P* values correspond to a Wald-based *F* test with denominator degrees of freedom estimated using the containment method.

Last, we did not find any significant differences across study groups for outpatient use of primary care services, medication prescribing patterns, participant refill adherence, body mass index, or self-reported physical activity (eTable 10 in Supplement 2). Types of antihypertensives prescribed were similar across the 3 groups (eTable 11 in Supplement 2). In addition, data on incidence of stroke and all-cause mortality during follow-up across groups were inconclusive (eTable 12 in Supplement 2).

## Discussion

In this cluster randomized clinical trial, we found that a culturally appropriate telephone-based lifestyle intervention (eg, one that incorporates photographs, stories, and recipes that are relatable to Black adults) using motivational interviewing skills and focused on the DASH eating plan improved BP control in Black adults with uncontrolled hypertension compared with UC, even as long as 3 years after the intervention ended. These results remained the same whether the primary outcome was assessed at a single time point or if all available BP data were used via sensitivity analyses.

Despite substantial improvement in BP control among KPNC patients over time,^14,29^ significant disparities remained in hypertension control between Black and White adults. Our study observed that the LC approach effectively helped Black adults with uncontrolled hypertension compared with UC, and this effect continued to 3 years past the end of assistance by LC coaches. Prior studies also supported that an in-person or a video education intervention was more successful than printed educational materials in achieving BP control.^30^ Our LC intervention combined motivational interviewing methods with an emphasis on the DASH eating plan with sodium reduction, which was previously shown to lower BP in Black patients with hypertension.^24,25^ Regular monitoring during the study was set up to promote adherence to the intervention protocols and the fidelity of the motivational interviewing method.^20^ In addition, study staff were trained for culturally responsive care and building trust and strengthening relationships between clinicians and participants. The training used the AIDET (Acknowledge, Introduce, Duration, Explanation, and Thank) tool. Previously reported lifestyle intervention trials aimed at improving BP control varied greatly in the intensity of the intervention and had limited assurance of fidelity of the intervention.^31,32,33^

Although there is a viable role for medication therapy adherence in BP control,^34^ it did not appear to be the explanation for our trial results. However, we did not have reliable information on granular dose adjustments within individual patients. The LC group did not have increased use of outpatient primary care services, better body mass index, or increased self-reported physical activity. Follow-up in previous studies was limited, and no study has demonstrated efficacy of the intervention on BP outcomes beyond 12 months. Interestingly, our LC intervention did not show significant benefit over UC at 12 months after enrollment but was clearly effective at improving BP control at 24 and 48 months after enrollment. Owing to the pragmatic design, assessment of BP during the 12-month follow-up may have been inadequate. Future pragmatic studies on hypertension should consider the use of home-based BP monitoring to increase the number of data points.^35^ In addition, the participants could have needed more time to implement their learnings. Our findings support the importance of laying the appropriate foundation for patients to understand and manage their own chronic condition as part of future implementation efforts.

In addition, participants receiving the LC intervention were more likely to have sustained BP control than those receiving UC. Furthermore, improved BP control was noted in participants completing even 6 LC sessions during a 12-month period. This finding suggests that a shorter duration of LC may be adequate in laying the necessary foundation for better BP management. Our pragmatic design leveraged existing infrastructures within KPNC. However, our LC intervention could be readily adapted for implementation in other integrated health care delivery systems with similar availability of an integrated EMR with comprehensive pharmacy data (eg, the Veterans Affairs medical system and other types of accountable care organizations).

### Limitations

Our study also has limitations. All participants were members of an integrated health care delivery system with pharmacy benefits and access to care, which may limit the generalizability of study results to other more fragmented practice settings. Importantly, KPNC membership is highly representative of the sociodemographic characteristics of the adult population statewide.^36^ Pregnant women with hypertension were typically treated by obstetrics and gynecology clinicians and were not included. Enrolled participants were generally older, so future studies should identify any specific needs of and potential barriers to recruitment of younger patients and plan for tailored strategies to address them. Our institutional review board’s requirement to obtain consent from those in the intervention groups but not the UC groups led to different enrollment rates in each group and potentially a selection bias of more motivated individuals who chose to participate in the intervention groups rather than the UC groups. We chose a dichotomous BP outcome because it mirrored the National Committee for Quality Assurance Healthcare Effectiveness Data and Information Set criteria for BP control and was clinically meaningful to organizations such as ours that followed these criteria. However, this method did not consider all available BP measurements. Therefore, we have performed sensitivity analyses using all BP measurements over time. Our LC intervention was designed with one-on-one telephone sessions. However, future research should explore adaptation of this intervention via telemedicine and in group settings to reach a larger population of patients. Given the pragmatic design and to reduce costs for the study, we relied on the availability of free BP check clinic visits and EMR data to identify study outcomes and did not mandate BP check at any specified times during follow-up. This led to varying lengths of time for final BP outcomes per participant. Although all BP clinic staff were trained on standardized BP measurement technique per KPNC protocol, these measurements may not be as accurate as those performed via standard research protocols. Last, we had a low response rate of several instruments (such as the Morisky Medication Adherence Scale, salt-intake questionnaire, and a fruit-vegetables screener) across all groups and were unable to examine their influence on BP control.

## Conclusions

In this cluster randomized clinical trial, we found that a culturally appropriate, telephone-based LC intervention was more successful than UC at improving BP control among Black adults with persistent uncontrolled hypertension. The LC intervention was feasible to implement in this high-risk population and was effective in helping these adults manage their chronic condition even 3 years after the intervention ended. Future research efforts should explore the implementation of this successful intervention in different clinical settings and populations.

## Notes

Supplement 1.
Trial Protocol

Supplement 2. **eMethods.** Treatment Groups and Statistical Analysis **eResults.** Sensitivity Analysis **eTable 1.** Distribution of Mean Blood Pressure at Baseline and 12, 24, and 48 Months Post Enrollment by Study Group **eTable 2.** Timing of Blood Pressure Measurements Carried Forward at Each Follow-up Period **eTable 3.** Blood Pressure Outcomes at 12, 24, and 48 Months Post Enrollment, Including 81 Additional Subjects Who Declined to Participate in EP (n = 26) or LC (n = 55) Group but Allowed EMR Data Use **eTable 4.** Blood Pressure Outcomes at 12, 24, and 48 Months Post Enrollment Adjusted for Physician Patient Panel Size **eTable 5.** Blood Pressure Outcomes at 12, 24, and 48 Months Post Enrollment Among Participants Who Had Available Blood Pressure Data Recorded During the Study Period **eTable 6.** Models of Blood Pressure Control at 12, 24, and 48 Months Post Enrollment Stratified by Sex and Age **eTable 7.** Blood Pressure Outcomes at 12, 24, and 48 Months Post Enrollment With Alternate Cut Point for Blood Pressure Control (<130/80) **eTable 8.** Blood Pressure Outcomes at 12, 24, and 48 Months Post Enrollment With Models Using Multiple Imputations **eTable 9.** Sensitivity Analysis for Blood Pressure Outcomes at 48 Months Post Enrollment **eTable 10.** Outcomes Across the Groups for Outpatient Utilization of Primary Care Services, Clinician Prescribing Pattern for Antihypertensives, Participant Compliance With Refills, Body Mass Index, or Self-reported Physical Activity **eTable 11.** Types of Antihypertensive Prescribed Across the 3 Groups **eTable 12.** Clinical Outcomes During Postintervention Follow-up **eFigure 1.** Probabilities of Controlled BP Over Time for Usual Care, EP Group, and LC Group Based on the Generalized Linear Mixed-Effect Model **eFigure 2.** Adherence to Lifestyle Coaching Sessions (n = 286)

Supplement 3.
Data Sharing Statement
