# Supplementary material for: Effect of Lifestyle Coaching or Enhanced Pharmacotherapy on Blood Pressure Control Among Black Adults With Persistent Uncontrolled Hypertension: A Cluster Randomized Clinical Trial
Source: JAMA Netw Open. 2022 May 18;5(5):e2212397. doi: 10.1001/jamanetworkopen.2022.12397 (PMC9118047; doi:10.1001/jamanetworkopen.2022.12397)
Supplement: Supplement 1. — Trial Protocol [file jamanetwopen-e2212397-s001.pdf]

SHAKE, RATTLE AND ROLL

Subtitle: Shake, Rattle and Roll is a pragmatic cluster randomized trial of 194 primary care provider panels to: 1) control or usual care; 2) diet & lifestyle coaching intervention; or 3) enhanced blood pressure protocol intervention.

Principal Investigator:

Mai N. Nguyen-Huynh, MD MAS

Kaiser Permanente Division of Research

2000 Broadway Ave., 5th floor

Oakland, CA 94612

Co-Principal Investigator:

Stephen Sidney, MD MPH

Kaiser Permanente Division of Research

2000 Broadway Ave., 5th floor

Oakland, CA 94612

Supported by:

The National Institute of Neurological

Disorders and Stroke (NINDS)

Version 1.4

09/13/2013

TABLE OF CONTENTS

1. STUDY OBJECTIVES ……6

1.1 Primary Objective ……6

1.2 Secondary Objectives ……6

2. BACKGROUND ……6

2.1 Rationale ……6

2.2 Supporting Data ……9

3. STUDY DESIGN ……15

4. SELECTION AND ENROLLMENT OF SUBJECTS ……18

4.1 Inclusion Criteria……18

4.2 Exclusion Criteria ……18

5. STUDY INTERVENTIONS ……18

5.1 Control Group……18

5.2 Diet & Lifestyle Coaching Intervention……19

5.3 Enhanced BP Protocol Intervention……21

6. CLINICAL AND LABORATORY EVALUATIONS ……22

6.1 Schedule of Evaluations ……22

6.2 Timing of Evaluations ……22

6.3 Recruitment and consent……23

6.4 Assessments: Surveys and Sodium Tests……23

6.5 Definitions of Evaluations……24

6.6 Medical History……26

6.7 Laboratory Evaluations……26

7. MANAGEMENT OF ADVERSE EXPERIENCES ……26

8. CRITERIA FOR INTERVENTION DISCONTINUATION ……26

9. STATISTICAL CONSIDERATIONS ……27

9.1 General Design Issues ………………………………………………………..…....….27

9.2 Outcomes ………………………………………………………………..…..…….28

9.3 Sample Size and Accrual ………………………………………………..……..…28

9.4 Data Monitoring ………………………………………………………..……......29

9.5 Data Analyses ………………………………………………………………..…..…..29

10. DATA COLLECTION, SITE MONITORING, AND ADVERSE EXPERIENCE REPORTING ………………………………………………………………………..........30

10.1 Records to be Kept ……………………………………………………………..…30

10.2 Role of Data Management ………………………………………………….…30

10.3 Quality Assurance …………………………………………………………………....31

10.4 Adverse Experience Reporting …………………………………………………..31

11. HUMAN SUBJECTS ………………………………………………………………..……32

11.1 Institutional Review Board (IRB) Review ………………………………......…..32

11.2 Subject Confidentiality ………………………………………………………...…..32

11.3 Study Modification/Discontinuation ……………………………………….……..…32

12. PUBLICATION OF RESEARCH FINDINGS ……………………………......……..32

13. REFERENCES ……………………………………………………………..…………33

Shake, Rattle & Roll Protocol Synopsis

Title

"Shake" the salt habit, "Rattle" the intensity of the current BP protocol, "Roll" out the interventions to community clinics.

Primary Objective

To determine whether a primary prevention intervention of either diet and lifestyle coaching or an enhanced pharmacotherapy protocol is more effective than usual care in improving rates of hypertension (HTN) control in blacks and thereby reducing disparities between black and white.

Secondary Objectives

To determine: 1) sustained blood pressure (BP) control at 2 and at 3 years post-enrollment; 2) a combined endpoint of stroke, major cardiovascular event, and death due to vascular causes; 3) each component of the combined endpoint; 4) time to BP control; and 5) percentage of time BP stays under control at 1-year, 2-year, and 3-year.

Study Design

A cluster randomized controlled trial at the primary care provider (PCP) level and including 194 PCPs within KPNC East Bay service area. The unit of randomization will be the clinician, with all adult African American members of each clinician's panel being the members of that clinician's cluster. We will randomize all PCP patient panels to a three-arm trial to receiving either 1) usual care; or 2) culturally tailored diet and lifestyle coaching with an emphasis on the DASH (Dietary Approaches to Stop Hypertension) diet; or 3) an enhanced BP management protocol with pharmacotherapy.

Patient Population

African American members of Kaiser Permanente with primary care providers in the East Bay Service Area who are 18 years or older with hypertension.

Inclusion/Exclusion Criteria

Inclusion:

- Kaiser Permanente member throughout the study period with pharmacy benefits
- Age ≥18
- In the hypertension registry
- Self-reported race of African American
- Sufficient understanding of the English language

Exclusion:

- Non-Kaiser Permanente member
- Age<18 or >85 years;
- A race other than African-American
- Not included in hypertension registry
- Pregnant women
- End-stage renal disease (ESRD);
- Dementia;
- Members in hospice or on home hospice or have life expectancy of <6 months
- Members in skilled nursing facility (SNF);
- Non-English speaking

**Randomization**	Randomization will be done within strata defined by the size of the clinician's panel of African American patients: 41 panels with 1-10 black patients, 34 with 11-50 black patients, 57 with 51-100 black patients, and 59 with more than 100 black patients, striving for an approximately equal number of African Americans in each arm (control: diet/lifestyle coaching: enhanced BP intervention)
**Primary Outcome**	The proportion of African American patients with hypertension who achieved BP control at 1-year post-study enrollment. BP control is defined as having a SBP<140 and DBP<90.
**Secondary Outcomes**	1) Combined outcome of stroke occurrence, vascular death, or myocardial infarction. 2) Vascular death. 3) Myocardial infarction. 4) Time to first BP control. 5) Proportion of time with BP under control. 6) Use of spironolactone. 7) Mean change of SBP and DBP. 8) Per protocol analysis of BP control at 1-year post-study enrollment.
**Interventions & Duration**	1) Control or usual care. 2) Diet & lifestyle coaching intervention. 3) Enhanced BP control protocol. Interventions will be for a duration of 12 months from enrollment. After the interventions ended, patients will continue to be followed for up to 3 years.
**Sample size**	A total of 5687 African Americans with uncontrolled HTN with approximately 1896 in each arm of the study.
**Primary Statistical Analysis**	The primary hypothesis will be tested by comparing between groups using a generalized linear mixed effects model (logit link) which will account for the within-cluster (clinician) correlation among patients.

.

1 STUDY OBJECTIVES

1.1 Primary Objective:

The primary research question of this study is to determine whether a primary prevention intervention of either diet and lifestyle coaching, or an enhanced pharmacotherapy protocol is more effective than usual care in improving rates of HTN control in blacks and thereby reducing disparities between African Americans and whites. The primary aim is to assess whether by implementing either intervention, we will reduce the disparity in HTN control rates between blacks and whites by 4% at 1 year post-study enrollment.

1.2 Secondary Objectives:

A number of secondary analyses will be performed including evaluation of:

- sustained blood pressure control at 2 and 3 years
- a combined endpoint of stroke, major cardiovascular event, and death due to vascular causes
- each of the individual outcomes within the combined endpoint
- time to blood pressure control
- percentage of time blood pressure remains under control at 1-year, 2-year, and 3-year follow-up post-enrollment
- mean change in systolic blood pressure (SBP) and diastolic blood pressure (DBP) during intervention;
- per protocol analysis of BP control at 1-year post-enrollment.

2 BACKGROUND

2.1 Rationale

The burden of stroke

There are approximately 795,000 new and recurrent strokes per year in the US, of which 87% are ischemic.[1] Stroke is the fourth most common cause of death in this country and the number one cause of adult long-term disability worldwide.[1] The estimated direct and indirect costs of stroke care for 2009 are $68.9 billion.[2] The prevalence of stroke and its cost will undoubtedly rise as the aging population increases.

Stroke patients are at high risk of recurrent stroke. It is estimated that there are 170,000 to 220,000 recurrent strokes per year,[3] with the highest risk of recurrence in the first year after stroke (12-13%).[4-7] The annual risk falls to 3-4% after the first year. The case fatality rates of recurrent stroke are twice those of first event.[4, 5] Programs that can effectively address risk factors for stroke would go a long way in its prevention. Disease burden and growing disparities among certain populations are characteristics of heart disease and stroke epidemic. Stroke incidence is about twice as high for blacks as it is for whites.[8, 9] Similarly, stroke mortality rate is also higher among blacks than whites. The ratio of disparity is most evident at younger ages, and then decreases with age so that blacks and whites have similar stroke mortality rates by age 80 years.[10, 11] The relative risk of stroke recurrence for blacks also appears to be higher than whites (relative risk = 2.0; 95% CI: 0.9-4.4).[12] For those aged 65-74 years old, blacks had higher rate stroke readmission rate than whites by 40% even after adjustment for patient and hospital factors (RR = 1.40; 95% CI: 1.19-1.64).[13]

Despite the severe economic burden of stroke care, research on quality improvement interventions in stroke care and stroke risk management has been limited. Most prior studies were non-randomized and included too few patients and/or institutions for appropriate analysis. The costs of those interventions were rarely assessed.

HTN and stroke

Hypertension (HTN), defined as blood pressure (BP) ≥140/90, is likely the most important modifiable risk factor for stroke, affecting >70 million American adults.[14] HTN is also a major risk factor for heart disease (the most common cause of death in the U.S.), end-stage renal disease, and peripheral vascular disease.[15] Approximately one in three adults in the U.S. has HTN; and the percentage affected is much higher with older

age, >70% in those over 75 years old.[14] HTN is also more prevalent among women than men, excluding pregnant women. The prevalence of HTN is substantially higher in blacks.[16, 17]

It is estimated that approximately 50% of new strokes could be eliminated by controlling HTN successfully.[18] HTN is a potent risk factor for cardiovascular disease and stroke, but one that is treatable. Multiple randomized trials have shown that treating HTN is important in primary and secondary stroke prevention. In a meta-analysis of 14 studies with 37,000 patients with HTN, a reduction of 5-6 mmHg in diastolic BP was associated with a 42% reduction in stroke risk.[19] In another 9 trials with 6752 patients with stroke, the risk reduction was 28% with anti-hypertensive therapy.[20] The optimal level of BP control is still debatable. The use of anti-hypertensive therapy in a randomized trial (PROGRESS) has been shown to reduce the risk of recurrent stroke significantly, even in stroke patients with relatively normal BPs.[21] Similarly, in another trial, stroke risk was lower in those randomized to ramipril even when HTN was not present.[22] The current American Heart Association guidelines for prevention of stroke in patients with ischemic stroke recommend anti-hypertensive therapy to achieve a normal BP of <120/80 mmHg.[23]

It is still controversial which class of anti-hypertensive is best for reducing stroke risk in general population. Two met-analyses reported more stroke risk reduction seen with diuretics (39-51%) compared to beta-blockers (25-29%), although better control of HTN was achieved in the former.[24, 25] In another study, the angiotensin converting enzyme, captopril, was not as effective as diuretic or beta-blocker, but BP reductions were smaller with captopril.[26] In the Syst-Eur trial, there was a 42% stroke risk reduction with a calcium-channel blocker.[27] Choice of anti-hypertensive therapy is probably less important than actual BP reduction.[28] Given the same level of BP reduction, some would argue that ACE inhibitors and angiotensin receptor blockers may provide greater benefit in stroke risk reduction than other anti-hypertensive agents.[21, 22, 29]
Despite the existence of effective therapy for >50 years, BP control is not achieved in most patients. In 2008, 90.6 percent of adults aged 18 years and older had their BP measured within the preceding 2 years and could state whether it was normal or high.[30] In 2005-2008, 70.4 percent of adults aged 18 years and older with HTN were taking the prescribed medications to lower their BP. The proportion of those with controlled BP varied by age group: 37.5% of those aged 18-44 years, 48.9% of those aged 45-64 years, and 45.6% of those aged ≥65 years. National health objectives 2020 include reducing the proportion of adults with HTN from 29.9% to 26.9% or a 10% improvement and raising the percentage of those with controlled BP from 43.7% to 61.2%%.[30]

Racial-ethnic disparities in HTN control

African Americans have significantly higher rates of cardiovascular mortality, stroke, HTN-related heart disease, congestive heart failure, type 2 diabetes mellitus, hypertensive nephropathy, and end-stage renal disease. HTN disproportionally affects African Americans compared with whites. From the National Health and Nutrition Examination Survey (NHANES) for 1999-2002, the age-adjusted prevalence of HTN was higher among non-Hispanic blacks (40.5%) than whites (27.4%) or Mexican Americans (25.1%). Blacks were more aware of having high BP (70.3%) than whites (62.9%) or Mexican Americans (49.8%).[31] However, among those who were treated, blacks were less likely to have their BP adequately controlled. During 1999-2004, 68% of African American men were aware of having high BP and 56% were receiving medications (which were similar to their white counterparts), but only 30% had their HTN controlled while 39% of White men achieved control.[32] A number of different studies on racial differences in treatment and control of HTN have yielded similar findings.

Prior proposed explanations for racial disparities in BP control include factors associated with biological, cultural, social, health care provider and health care system.[17, 33, 34] Some other proposed barriers include poor access to healthcare services and medications, insufficient attention by healthcare providers, lack of appropriate resources to make recommended lifestyle changes, and compliance with medications.

The current consensus statement from the HTN in African Americans Working Group of the International Society on HTN in Blacks highlighted 3 major paradigm shifts in treating high BP that are applicable to African Americans: "1) urging and supporting therapeutic lifestyle changes; 2) conducting a thorough cardiovascular risk assessment; and 3) achieving and maintaining a target BP that is determined by the individual's level of risk."[35]

Rationale for an individualized diet and lifestyle coaching intervention

Knowledge, beliefs, and attitudes about HTN among African Americans can affect health behaviors, perceptions of susceptibility to HTN, and adherence to treatment plan.[36] Patients' awareness and knowledge of prevention measures could have a significant role in adherence to management. Although physician education and practice are additional barriers, there is evidence to suggest that more educated and motivated patients are important instigators of improved physician adherence to quality improvement efforts.

Therapeutic lifestyle changes such as weight maintenance or reduction, increased physical activity, moderation of salt and alcohol intake and tobacco avoidance can lower BP.[37-39] For African Americans, obesity, high sodium and low potassium intake, and inadequate physical activity have been identified as particular obstacles to cardiovascular health.[40-42] The Dietary Approaches to Stop Hypertension (DASH) diet was found to be particularly beneficial in lowering BP in African Americans.[15, 43] This is a diet rich in fruits, vegetables, fiber, and low-fat dairy but with reduced saturated and total fats. The DASH diet with sodium restriction was found to confer additional benefit in lowering BP and was also more effective in African Americans than in others.[43] The International Society on HTN in Blacks recommended that plans for diet and exercise should be initiated in the primary care setting; and "recommendations should be specific, individually tailored, and well supported with counseling efforts and effective patient education materials."[35] On-going education and support play an important role in sustained changes in health behaviors.

By targeting blacks with uncontrolled HTN for an individualized wellness coaching program to promote physical activity and appropriate diet with an emphasis on the DASH diet using brief negotiation skills and motivational interviewing techniques and culturally tailored education materials, a higher rate of HTN control may be achieved among African Americans.

Theoretical basis for behavior change

Previous research suggests that people's health beliefs have great impact on their preventative health behavior.[44] A prominent model that has been proposed to account for this relationship is the Health Belief Model (HBM).[45, 46] The model is based on the understanding that a person will take a health-related action if: 1) he/she perceives that a bad health outcome can be avoided; 2) has a positive expectation that by following the recommended action, he/she will avoid this bad outcome; and 3) believes that he/she can carry out the recommendation. The original model included four constructs: 1) perceived susceptibility ("am I at risk for this disease?"); 2) perceived severity ("how serious is this?"); 3) perceived barriers; and 4) perceived benefits.[47] A number of mediating factors were later added to connect these perceptions with the predicted health behavior. These included demographics, socio-psychological variables, perceived self-efficacy (assessment of whether one can successfully adopt the desired behavior), health motivation, perceived control and perceived threat, and cues to action (external influences such as the media, reminders from primary care physicians, educational programs, etc.; diagram below).

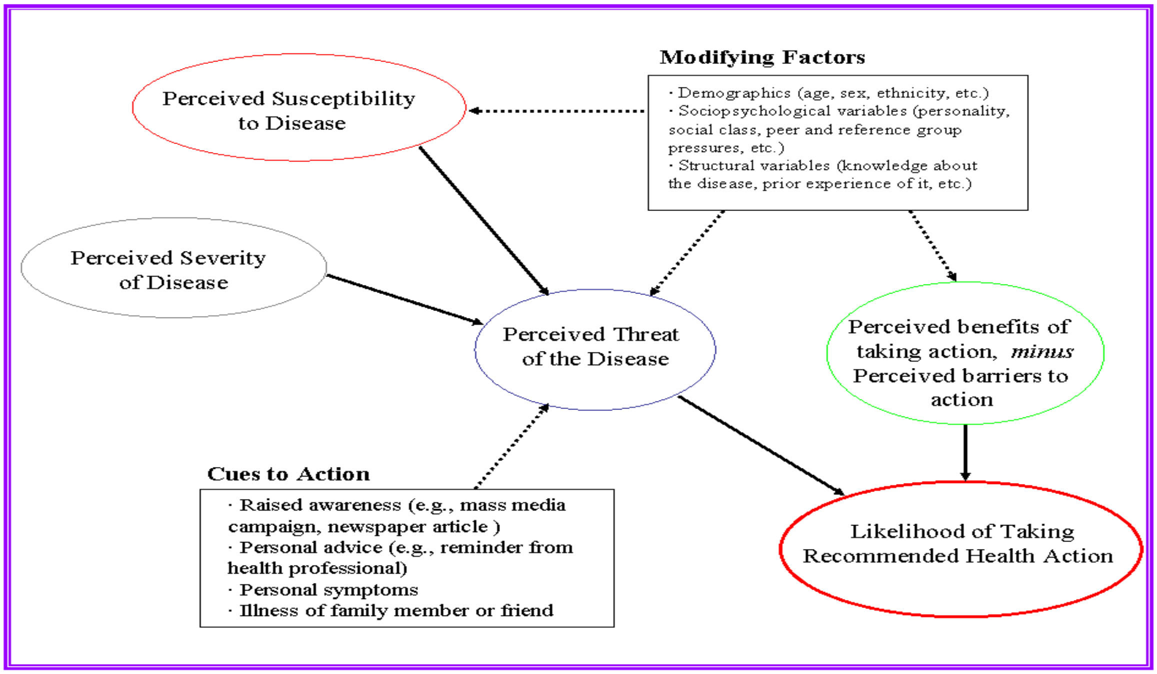

The HBM has been applied in areas related to stroke such as coronary heart disease prevention.[48, 49] More recently, researchers have explored the role of HBM beliefs in stroke prevention.[50-52] One study specifically used HBM to identify variables that may change in response to education.[52] The authors tested a simple education intervention of standard (educational brochure plus activities that were not about stroke) versus enhanced (educational brochure plus activities designed to enhance beliefs about stroke). They found that selected beliefs (perceived susceptibility and perceived benefit) changed significantly over time. Their findings suggest that basic approaches to patient education may influence health beliefs. Such beliefs could predict intention to change behavior. More effective HTN control programs may result from consideration of the role of health beliefs in such programs.

Our diet and lifestyle coaching intervention makes use of the concepts from HBM to test the influence of an individualized coaching program on HTN control and on patient utilization and adherence of evidence-based BP control therapies.[47] The coaching program will raise patients' awareness of HTN and its role as a major risk factor for stroke and cardiovascular disease. The coaching will also provide "cues to action" by raising awareness about low-salt (DASH) diet and active lifestyle. In addition, the intervention will also help the patients identify barriers to implementation of healthy behaviors and ways to overcome these barriers. The study hypothesis is that the intervention group would consequently acquire greater awareness of their risks, the possibility of their serious effects, and the benefits of the treatment plans through personalized coaching sessions compared to the control group who will receive usual care. We further hypothesized that this increased level of awareness would lead to a higher rate of treatment adherence and better HTN control. Our intervention is designed to increase patients' perceived susceptibility by increasing their knowledge of HTN, adopting healthy lifestyle changes and helping them to overcome individual-specific barriers to treatment adherence.

Rationale for a pharmacologic BP management protocol

Barriers to normalizing BP in African Americans have been largely attributed to biologic and social factors, with an inadequate emphasis on medical management. A study on physician-related barriers to effective management of uncontrolled HTN found the key obstacle to be the failure of medical providers to treat high BP early and to continue to treat it persistently in order to maintain appropriate BP goals.[53]

It has been reported that blacks may be responsive to different pharmacologic treatment of HTN.[54] The Antihypertensive and Lipid-lowering Treatment to Prevent Heart Attack Trial (ALLHAT),[28] in which 36% of the subjects were African American, found thiazide-type diuretics (chlorthalidone) to be superior to calcium channel blocker and angiotensin-converting enzyme (ACE) inhibitor for prevention of major coronary events.

Similar to other studies, blacks in ALLHAT were observed to have a poorer response in BP control with ACE-inhibitor.[55-57] Studies have also shown that blacks achieved better BP control with a calcium channel blocker than a beta-blocker.[55, 57] As monotherapy, thiazide diuretics and calcium channel blockers have greater BP lowering effect in African Americans than do other drug classes.[55, 58] However, large randomized clinical trials have found that typically 2-4 antihypertensives are required to control diastolic and systolic BP in adults with uncomplicated HTN.[26-28, 59-61] African Americans with HTN will usually require at least 2 medications to control BP. It is generally preferable to combine low doses of 2 antihypertensives than increasing the dose of the first agent.[58, 62, 63] The following combination therapy are considered effective in lowering BP: beta-blocker/diuretic[64]; ACE inhibitor/diuretic[65]; ACE inhibitor/calcium channel blocker[62]; or angiotensin II receptor blocker (ARB)/diuretic[66-68]. A major and often overlooked drug class that is typically used as add-on drug therapy is the aldosterone antagonists which are highly effective BP-lowering agents in African Americans. Spironolactone is the prototypical agent in this class that has been used to dramatically lower BP in patients with resistant HTN with and without primary aldosteronism.[69]

In the proposed intervention for an enhanced BP management protocol, we will target African Americans with uncontrolled BP aggressively and provide treatment persistently using evidence-based effective pharmacotherapy regimens for African Americans until HTN control is achieved.

Significance

The proposed project will assess 2 interventions carefully thought out, culturally-tailored and designed as a response to the consensus statement from the HTN in African Americans Working Group of the International Society on HTN in Blacks. By targeting African Americans with uncontrolled HTN, we are targeting the highest risk group for cardiovascular disease and stroke. By including young adult African Americans in our interventions, we are reaching out to improve HTN control rates in an often-overlooked population which carries the biggest disparities between black and white and therefore potentially will provide us with the greatest impact in reducing disparities. We have designed an individualized diet and lifestyle telephone coaching program that provides ongoing support and helps to identify barriers specific to African Americans with HTN, focuses on culturally appropriate education materials including the DASH diet, accommodates the demands of working individuals and accounts for the inconvenience of traveling. The second intervention proposed is an enhanced BP management protocol with pharmacotherapy. This approach modifies the currently highly effective BP control protocol in Kaiser Permanente Northern California (KPNC) in order to target African Americans with uncontrolled BP earlier and treat more aggressively with medications that have been shown to have the greatest benefit in BP reduction in blacks. Again, this intervention will also target young adult African Americans with uncontrolled BP, a group which has not received as much aggressive HTN management. A cost-effective intervention identified here could motivate other healthcare systems and facilitate the translation and dissemination of a HTN control program in the communities.

2.2 Supporting Data

Study on effect of race-ethnicity on BP control 6-month post stroke in KPNC

A retrospective cohort study of patients with discharge diagnosis of acute ischemic stroke was included in a randomized quality improvement trial (QUISP)[70] focused on secondary stroke prevention, conducted in 14 KPNC medical centers between January 2004 and September 2006. Patient characteristics, diagnoses and health care utilization data were gathered from administrative and clinical databases, including all anti-hypertensives prescribed at discharge and within 6 months post-discharge, post-discharge visits attended, medication adherence, and BP taken at 6 ± 2 months. Self-reported race-ethnicity data were available from inpatient administrative databases. HTN was defined as having either a history or a discharge diagnosis of HTN, or receiving anti-hypertensives at discharge. Compliance with prescriptions was determined by examining specific medication orders and filled prescriptions. Physician aggressiveness in treating HTN was assessed by examining the number of prescribed medications.

We abstracted detailed information on socio-economic status including type of insurance, types of pharmacy coverage, median household income, marital status, number of dependents covered under the same insurance plan, and living arrangement. We also ascertained information on scheduled clinic visits within

the first 6 months post-discharge. The primary outcome measure was optimal BP control, an average systolic BP <140 and diastolic BP <90 for measurements at 6 ± 2 months after hospital discharge for acute ischemic stroke. We examined race as a predictor of anti-hypertensive therapy, compliance with prescriptions, follow-up visits, and BP control (<140/90 mmHg) at 6 months after admission among all patients with HTN, adjusted for socio-economic status, age, gender, and dementia.

Data were obtained for 3172 patients with ischemic stroke. (A partial Table 1 with cohort characteristics is provided below.) Of these, 2972 (93.7%) had HTN; 52% were female. Average age was 73.2 ±12.2 years. Racial distribution was 66% non-Hispanic white, 14% African-American, 11% Asian, 8% Hispanic, and 1% other. Compared to non-Hispanic whites, African American, Asian, and Hispanic stroke patients were younger, less likely to be widowed and more likely to live with family at time of admission (Table 1). At least 96% of the patients had pharmacy coverage regardless of racial origin. Clinically, blacks had higher body mass index (BMI), and higher rates of diabetes and current cigarette smoking compared to whites (Table 2). At discharge, African American patients had higher mean BP (systolic 141 ± 19.6, diastolic 78 ± 16m7 mmHg) than whites (systolic 139 ± 19, diastolic 74 ± 13 mmHg), and were also prescribed more anti-hypertensives.

Characteristics of patients with ischemic stroke by races

Variable	White	Black	Asian	Hispanic	Other	p-value
Total n=2972 (93.7%)	n= 1955	n=427	n=324	n=227	n=39	
**From discharge to 6 months post stroke:**						
On at least one anti-hypertensive	1596 (81.6)	368 (86.2)	276 (85.2)	200 (88.1)	35 (89.7)	0.016
Average number of different antihypertensives (± SD)	2.2 (± 1.2)	2.5 (± 1.3)	2.3 (± 1.2)	2.3 (± 1.2)	2.1 (± 1.3)	<0.001
Types of antihypertensive prescribed:						
ACE-inhibitor	789 (40.4)	212 (49.7)	165 (50.9)	133 (58.6)	14 (35.9)	<0.001
Angiotensin receptor blocker	166 (8.5)	44 (10.3)	43 (13.3)	22 (9.7)	6 (15.4)	0.048
Beta-blocker	1037 (53.0)	225 (52.7)	191 (58.9)	131 (57.7)	22 (56.41)	0.235
Calcium channel blocker	430 (22)	142 (33.3)	76 (23.5)	47 (20.7)	11 (28.2)	<0.001
Diuretic	738 (37.8)	199 (46.6)	105 (32.4)	78 (34.4)	14 (35.9)	0.001
Other (vasodilator, alpha-blocker)	229 (11.7)	65 (15.22)	36 (11.1)	34 (15)	4 (10.3)	0.197
Follow-up visits:						0.229
None	341 (17.4)	56 (13.1)	45 (13.9)	31 (13.7)	5 (12.8)	
1 visit	416 (21.3)	86 (20.1)	68 (21)	43 (18.9)	10 (25.6)	
2 visits	1198 (61.3)	285 (66.7)	211 (65.1)	153 (67.4)	24 (61.5)	
**6 months post stroke:**						
Blood pressure <140/90	1199 (61.4)	225 (52.7)	205 (63.3)	134 (59.0)	26 (66.7)	0.01
On at least one anti-hypertensive	1234 (77.3)	268 (72.8)	222 (80.4)	157 (78.5)	23 (65.7)	0.08
Average number of different antihypertensives (± SD)	1.7 (± 1.2)	2 (± 1.4)	1.9 (± 1.1)	1.9 (± 1.2)	1.5 (± 1.3)	0.043

At 6 months, BP was controlled in 58% of hypertensive patients. African-Americans were not different from whites in attendance at outpatient visits or number of anti-hypertensives prescribed or compliance with prescriptions; however, they were significantly less likely to have their HTN controlled at 6 months when compared to whites (52.7% compared to 61.4%; OR=0.63, 95% CI 0.48-0.82, p=0.001). In multivariable analysis, having pharmacy coverage, dementia, and attending follow-up visits were independently associated with better BP control. On the other hand, being female (p=0.005), black (p=0.001), and having a higher number of different types of anti-hypertensives prescribed were independently associated with poorer BP control. Although socio-economic variables and BMI varied significantly among races, these did not change

the effect of race on BP control.

Table 2 Factors that influence whether blood pressure is under control (<140/90 mmHg) at 6 months post-stroke

	Univariate Analysis*			Multivariable Analysis*		
Patient characteristic	O.R.	95% C.I.	p-value	O.R.	95% C.I.	p-value
Age (decade)	0.85	(0.80 - 0.91)	<0.001	0.95	(0.86 - 1.04)	0.271
Gender (female vs. male)	0.78	(0.68 - 0.91)	0.001	0.75	(0.62 - 0.92)	0.005
Race/ethnicity (white as ref)						
African American	0.72	(0.57 - 0.90)	0.004	0.63	(0.48 - 0.82)	0.001
Asian/Pacific Islander	1.09	(0.85 - 1.39)	0.496	0.95	(0.70 - 1.30)	0.768
Hispanic	0.9	(0.68 - 1.20)	0.486	0.77	(0.55 - 1.08)	0.128
Marital status (single as ref)						
Married	1.22	(0.89 - 1.67)	0.29	1.36	(0.93 - 1.98)	0.11
Divorced	1.06	(0.71 - 1.58)	0.773	1.54	(0.94 - 2.51)	0.086
Widowed	0.96	(0.69 - 1.34)	0.819	1.51	(0.98 - 2.33)	0.059
Median household income (by $10,000)	1.03	(1.00 - 1.07)	0.042	1	(.99 - 1.00)	0.611
Has pharmacy coverage	1.83	(1.13 - 2.97)	0.014	2.14	(1.1 - 4.19)	0.025
Mean BMI	1.01	(0.99 - 1.02)	0.156	1.01	(0.99 - 1.03)	0.194
Dementia	0.93	(0.73 - 1.18)	0.534	1.46	(1.02 - 2.09)	0.038
Current cigarette smoking	0.95	(0.82 - 1.11)	0.538	1.02	(0.81 - 1.27)	0.88
Current alcohol use	1.07	(0.95 - 1.22)	0.269	1.18	(0.98 - 1.43)	0.076
On any anti-hypertensive during study	1.32	(1.10 - 1.61)	0.004	0.97	(0.70 - 1.34)	0.876
Number of different types of BP** med during study	0.83	(0.78 - 0.89)	<0.001	0.74	(0.63 - 0.88)	<0.001
Total number of anti-hypertensives during study	0.92	(0.86 - 0.98)	<0.001	1.05	(0.87 - 1.26)	0.607
Post-discharge clinic visits (0 as ref)						
1	3.33	(2.58 - 4.30)	<0.001	3.99	(2.77 - 5.76)	<0.001
2	5.7	(4.56 - 7.12)	<0.001	6.91	(4.92 - 9.70)	<0.001

Our study found that African Americans with ischemic stroke had poorer BP control at 6 months post-discharge compared to whites despite having equal healthcare utilization, prescriptions for anti-hypertensive, and compliance with filling prescriptions. The finding remained robust to changes in key model assumptions. Differences in socio-economic status, co-morbidities, BMI, and exact BP measurements at discharge did not significantly explain this disparity in HTN control. Differences in severity of HTN and lifestyle/diet may account for these findings, but were not readily available for assessment in this study.

PHASE (Preventing Heart Attack and Stroke Everyday) program in Kaiser Permanente

In 2003, Kaiser Permanente developed ALL (Aspirin, Lisinopril, and Lipid-lowering Medication) to reduce cardiovascular risk by aggressively enrolling patients >50 years old with diabetes in a therapeutic program using a triad of medications. In 2004, KPNC launched a region-wide project called Preventing Heart Attacks and Stroke Everyday (PHASE) to enhance ALL by adding a beta-blocker. PHASE is a population-based approach to ensure comprehensive secondary prevention of atherosclerosis in patients with any history of coronary artery disease (CAD), diabetes, stroke/transient ischemic attack (TIA), peripheral arterial disease (PAD), or abdominal aortic aneurysm (AAA). PHASE seeks to ensure that all these patients receive proven preventive medications and aggressive risk factor management to help slow the atherosclerotic process and prevent cardiac and cerebrovascular events. The clinical objectives encompass three components: 1) prescribe appropriate medications unless patient is allergic, intolerant or refuses (aspirin, statins, ACE inhibitors, beta-blockers); 2) achieve control of BP, lipids and glycemia for diabetes; and 3) advise lifestyle changes including tobacco cessation, physical activity, healthy eating, and weight management.

Inclusion criteria for PHASE registry are listed below. Detailed inclusion and exclusion criteria for the other registries (diabetes, CAD, stroke/CVA) are included in Appendix B.

PHASE Registry
**Inclusion Criteria**<br>Members who meet any of the following criteria within the last **36 months** are included in the PHASE registry:<br>• on the Diabetes registry<br>• on the CAD registry<br>• one or more stroke/CVA diagnosis in the clinic, ED or hospital setting<br>• one or more Abdominal Aortic Aneurysm (AAA) diagnosis in the clinic, ED or hospital setting<br>• one or more Peripheral Vascular Disease (PVD) diagnosis in the clinic (Optometry & Ophthalmology departments are excluded) ED or hospital setting<br>• Chronic Kidney Disease, defined for PHASE as age ≥50 and GFR <30 OR age ≥50 and GFR 30-59 with positive proteinuria result (excluding those who tested positive for nitrite and esterase on the same day)<br>• ESRD members are excluded from the PHASE registry

About 300,000 patients or 12% of KPNC adult membership are meet PHASE criteria. Because this is a secondary prevention program, approximately 84% of PHASE patients are ≥ 50 years old, and 50% are ≥65. Quality and Operations Support (QOS) produces a monitoring report for the PHASE population and also maintains the Population Management Tool (PMT) database. PMT database is available to all facilities and allows users to identify patients who are missing recommended office visits or lab tests. It is used for outreach efforts.

KPNC developed the HTN Registry in 2001. Members who meet at least one of the following criteria are included in the KPNC HTN registry:

- 2 or more primary care HTN diagnosis
- 1 or more primary care HTN diagnosis and 1 or more hospitalizations with a primary or secondary HTN diagnosis
- 1 or more primary care HTN diagnosis and 1 or more filled prescriptions for HTN medication within the last 6 months
- 1 or more primary care HTN diagnosis AND (1 or more stroke-related hospitalizations OR history of CAD, heart failure of diabetes)

An evidence-based BP management protocol has been developed by clinicians with expertise in HTN control and has been in effect for all of KPNC as part of PHASE since 2004. BP check visits were available starting around 2005 and are open to all patients whether they are in the HTN registry or not. These visits are free of charge to all members. A patient contact is initiated only if a risk factor is not under control (for example, BP>140/90) or if the patient is not on one of the four medications mentioned above. A snapshot of the BP protocol in PHASE is attached below. A full copy of the PHASE pocket card is in Appendix C.

Cardiovascular Risk Management **POCKET VERSION**

Medication Algorithm†

Plus Lab Chart and Simvastatin Conversion and Drug Interaction Table

PHASE

PREVENT HEART ATTACKS AND STROKES EVERYDAY

PHASE POPULATIONS

- **AAA**
- **CAD**
- **CVA/TIA** Ischemic, Embolic
- **PAD**
- **DM** ASA w/RF M ≥ 50 yrs, F ≥ 60 yrs, ACEI ≥ 55 yrs; Statin ≥ 40 yrs

PHASE MEDICATIONS & CAUTIONS

INDEPENDENT OF BP/LDL CONTROL

ASA

ASA	81mg daily

CAUTION/INFO If intolerant to ASA, consider Clopidogrel in CAD, PAD. If embolic CVA/TIA, warfarin preferred.

ACEI

Lisinopril	10mg daily

CAUTION/INFO Verify effective contraception in women of childbearing potential: use Chlorthalidone or HCTZ. Use caution with ACEI if eGFR <30 or K>5.5. Use ARBs if ACEI intolerant & HTN not controlled on thiazide alone.

ACEI+HCTZ – FOR HX ISCHEMIC & EMBOLIC CVA (& FOR INTRACRANIAL HEMORRHAGE)

Lisinopril/HCTZ	10–12.5mg daily

No special caution or info statement.

STATIN

Simvastatin	40mg daily

CAUTION/INFO Verify effective contraception in women of childbearing potential. If eGFR <30, reduce initial dose. Max Simvastatin dose 40mg daily. Use Atorvastatin if higher dose statin needed. Follow Simvastatin Conversion & DDI Table.

BETA BLOCKER – FOR CAD/PAD/AAA

Atenolol	25mg daily

CAUTION/INFO Use caution in bradycardia <55, severe asthma, hypotension. Alternative if HF or LVEF <40%, use Carvedilol or Bisoprolol. If eGFR <30, use Metoprolol.

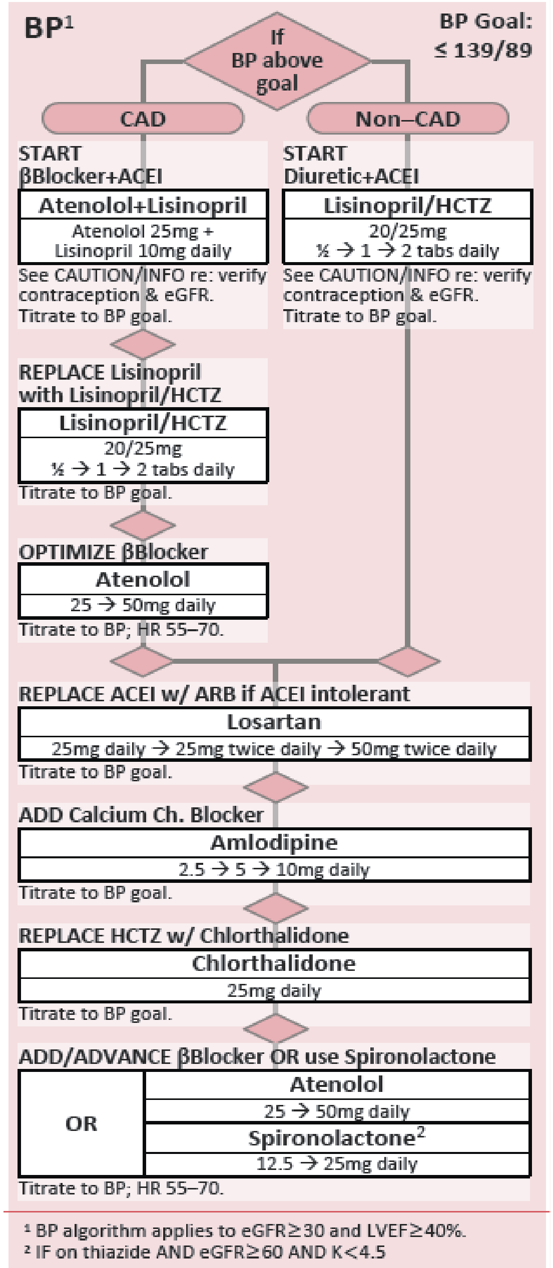

Current HTN control in KPNC

With a more structured BP control guideline, HTN control rates have improved significantly within KPNC (as well as across Kaiser nationally). For the 4th quarter of 2004, overall regional HTN control rate for the PHASE population (ages 18-75) was 44.3%. That rate increased to 64.1% by the 4th quarter of 2009, representing a 19.8% improvement. As HTN control rates improve in KPNC, there remains a clear disparity in

BP control between blacks and whites. From 4th quarter in 2009 through the 3rd quarter in 2010, BP control disparity between blacks and whites was reduced by 1.4%. However, for the immediate past year, from 2010 Q4 to 2011 Q3, the disparity has only been reduced by 0.6%. Although KPNC's BP control rates are significantly higher than the national average (which is roughly 50%), there is still room for further improvement including reducing racial-ethnic disparity.

BP management in KPNC East Bay Service Area

Approximately one-third of all KPNC African American members with HTN reside within the East Bay Service Area (EBSA). They typically receive medical care from 4 main centers: Oakland, Richmond, Pinole and Alameda. Currently there are >45,000 members in the HTN registry in the EBSA of which approximately 15,000 are black. Based on the latest data available for HTN control in EBSA, blacks have a 77.2% control rate versus 81.8% in whites (Table 1).

Table 1. Rates of BP control <140/90 (period ending September 30, 2010)

	**All members**	**Black or African American (n=9960)**	**Asian or Pacific Islander (n=5020)**	**Hispanic or Latino (n=3431)**	**Multiracial (n=617)**	**White (n=9521)**	**HEDIS National 90th percentile**
**Region (n=326,404)**	80.5%	76.1%	82.8%	79.7%	79.7%	80.9%	72.2%
**East Bay (n=28,631)**	80.9%	77.2%	85.2%	82.7%	80.5%	81.8%	72.2%

Preliminary data from first quarter 2012 showed that the greatest disparity in HTN control between black and white in the EBSA lies with the young adults <45 years of age (Table 2).

Table 2. HTN control rates by race and age groups.

	Black or African American			White		
Age (years)	Uncontrolled HTN (N)	Total with HTN (N)	Rate of uncontrolled (%)	Uncontrolled HTN (N)	Total with HTN (N)	Rate of uncontrolled (%)
<45	487	1604	**30.4**	337	1644	**20.5**
45-64	1803	7972	22.6	1379	8518	16.2
65+	966	5494	17.6	1171	8266	14.2
Total	3256	15609	20.9	2887	18428	15.7

When exactly a patient is seen for BP management after having an uncontrolled BP measurement recorded varies from medical center to center. Although it is recommended that patients with poor BP control and resistant HTN be seen more frequently and treated aggressively, it is not yet the current daily practice. In the EBSA, only 26.3% of the patients with resistant HTN, defined as being on 3 or more anti-hypertensives, were seen within 14 days of their last uncontrolled BP measurement. Approximately 75% were seen within 3 months of the last recorded uncontrolled BP measurement.

In addition, spironolactone has been shown to be highly effective in lowering BP in blacks with resistant HTN. It is currently rarely utilized although it is part of the BP management protocol in KPNC (Table 3).

Table 3. Rates of spironolactone prescription in patients with uncontrolled HTN (first quarter 2012)

Race-ethnicity	On ≥3 anti-hypertensives (N)	On ≥3 anti-hypertensives including spironolactone (%)	On any anti-hypertensive (N)	On any anti-hypertensive including spironolactone (%)
Asian or Pacific Islander	159	3.8	766	0.9
Black or African American	793	2.9	2543	1.5
Hispanic or Latino	5	0	35	0
Native American	5	0	26	0
Unknown	108	1.9	603	0.7
White	511	2	2252	1.1
Total	1581	2.6	6225	1.2

Significance of Prior Research and Proposed Interventions

Our prior work has demonstrated that despite overall improvement in HTN control rates over the past 8 years in KPNC, significant disparities remain in HTN control between black and white despite having similar access to care. In our study on the effect of race in BP control 6-month post-stroke, we found that blacks were seen for follow-up more often after stroke discharge, placed on more anti-hypertensives, were equally adherent to filling these prescriptions as other races, but remained with poorer BP control at 6 months. Greater difficulty in controlling BP and different lifestyles may explain this disparity. HTN management programs may not adequately account for racial differences in response to anti-hypertensive medications. Furthermore, the BP protocol is for the management of any patients with HTN, whether in PHASE program or not, but it is the PHASE population with chronic conditions that is targeted for care much more frequently than those without. In order to improve the disparity of HTN control between black and white, a population-based approach is necessary to target high risk black patients with uncontrolled BP quickly, often, and aggressively. The approach needs to also reach out to the young adult black population which is where the greatest impact in disparity may be achieved. The Institute of Medicine has made system change the main tenet of closing the “quality chasm.” Our proposal addresses the important challenges of treating high BP in African Americans by testing both lifestyle modifications as well as a pharmacotherapy approach as emphasized by the International Society on HTN in Blacks, and we will do so by using an integrated electronic medical record system to track co-morbidities, BP measurements, labs, and pharmacy data and providing wellness coaches and clinical pharmacists the skills necessary to deliver culturally appropriate care and behavioral counseling.

3. STUDY DESIGN

The “**Shake, Rattle and Roll**” trial is named for: 1) “shake” the salt habit; 2) “rattle” the intensity of the current clinical BP protocol; and 3) design the interventions with the ultimate goal of being able to adapt and “roll” out the interventions to community clinics outside of the managed care system.

This is a pragmatic cluster randomized controlled comparative effectiveness trial with randomization at the primary care provider level. The PCP patient panels will be randomized to one of 3 arms: 1) usual care; 2) diet and lifestyle coaching program; or 3) enhanced BP management with pharmacotherapy. The interventions will focus on African Americans with uncontrolled HTN (Figure– study flow diagram). We will identify eligible patients with uncontrolled HTN (defined as BP <140/90) using KPNC electronic databases and the HTN registry. Those who are randomized to the diet and lifestyle coaching intervention and opted to be in the study will be contacted by a trained wellness coach at regular intervals via telephone sessions as well as attending group education sessions (Figure – study flow diagram, below). Those who are randomized to the quality improvement effort utilizing an enhanced BP protocol will be contacted by a research coordinator to facilitate their BP check visits as needed and their medication regimens will be reviewed and modified as necessary by

a clinical pharmacist. All research staff and providers will be trained in cultural competency and tailoring interventions for African Americans. Both interventions will be implemented for a one-year duration post-enrollment.

The primary goal is to reduce HTN control disparity between blacks and whites by 4% at the end of 1 year. Primary outcome will be the proportion of African Americans with HTN who achieve BP control as assessed at the end of 1 year. Data for BP and clinical outcomes will continue to be collected for 36 months post-enrollment for secondary outcomes which will include BP control rates at 2 and 3-year post-enrollment, stroke/vascular death/myocardial infarction risk, time to BP control, and time stayed in BP control. Data will be analyzed by intention-to-treat. Demographics, adherence to prescriptions, socioeconomic and behavioral factors that may be related to success of the interventions will be examined.

Randomizing 194 PCP patient panels in KPNC East Bay Service Area

Control	Diet/lifestyle coaching	Intensified BP protocol
**Control (N= initial 1085 blacks with uncontrolled HTN + 375 new uncontrolled per year = 1460)**	**Diet/lifestyle coaching (N= initial 1085 blacks with uncontrolled HTN + 375 new uncontrolled per year = 1460)**	**Intensified BP protocol (N= initial 1085 blacks with uncontrolled HTN + 375 new uncontrolled per year = 1460)**
Send a letter to all African American members welcoming them to the HTN control program for blacks in KPNC. Include handouts on HTN control and low-salt diet.	Send a letter to all African American members welcoming them to the HTN control program for blacks in KPNC. Include handouts on HTN control and low-salt diet. And, inform them of the wellness coaching sessions and support, and additional education materials. Include a post-card for opt-out option.	Send a letter to all African American members welcoming them to the HTN control program for blacks in KPNC. Include handouts on HTN control and low-salt diet. And, emphasize the importance of attending the BP check clinic promptly as scheduled if indicated for them when their BP is out of control.
	5-minute pre-study diet screener for fruits/vegetables/grains via online or by phone for all participants	
Pre-study questionnaires mail out to random 400 participants: Stroke knowledge, 8-item modified Morisky scale, Salt intake.	Pre-study questionnaires mail out to random 400 participants: Stroke knowledge, 8-item modified Morisky scale, Salt intake.	Pre-study questionnaires mail out to random 400 participants: Stroke knowledge, 8-item modified Morisky scale, Salt intake.
pre-study 24-hr urine Na+ kit send out to a random sample of 100 participants	pre-study 24-hr urine Na+ kit send out to a random sample of 100 participants	pre-study 24-hr urine Na+ kit send out to a random sample of 100 participants
Continue with usual care	Schedule group classes with Health ED on HTN management and healthy lifestyle. Cuturally tailored education materials will be handed out at these sessions.	1) Analyst generates weekly list of patients with uncontrolled BP, and gives list of black members in this group to Research Coordinator who will contact members to set up appointment for BP check in 2-3 weeks from last uncontrolled BP measurements. 2) Research Coordinator will send message to MA groups and study clinical pharmacist to inform them of scheduled appointments for these partipants. 3) RA will contact patients to remind them of appointments.
	Wellness coaching through telephone sessions once a month for the first 3 months after enrollment.	At each BP check visit, the MA will route charts of black patients in this intervention group to the Research Coordinator and to the study clinical pharmacist who will make treatment recommendations for BP management.
	After the initial 3 months: 1) if BP is under control, continue with telephone coaching sessions once every 3 months; 2) if BP is not under control, monthly coaching sessions until BP is under control; 3) if at any time during the study BP becomes out of control, will resume monthly coaching	1) If at the BP check visit, BP is still not under control, the Research Coordinator will schedule the patient for another re-check appointment within 2-3 weeks. 2) RA will contact patients to remind them of appointments. 3) If BP is under control at visit, no follow up appointment. 4) If at any time during the study, BP becomes out of control, patient will be picked up in the analyst's weekly list of uncontrolled BP.
	mid-study 24-hr urine Na+ kit send out to the same 100 participants sometimes in the middle of their year in the study	
Continue until patient completes 1 year after enrollment	Continue until patient completes 1 year after enrollment	Continue until patient completes 1 year after enrollment
post-study 24-hr urine Na+ kit send out to the same 100 participants at the end of the study	post-study 24-hr urine Na+ kit send out to the same 100 participants at the end of the study	post-study 24-hr urine Na+ kit send out to the same 100 participants at the end of the study
At 1 year ± 1 month after enrollment: post-study questionnaires to the same random 400 participants: Stroke knowledge, 8-item modified Morisky scale, Salt intake.	At 1 year ± 1 month after enrollment: post-study questionnaires to the same random 400 participants: Stroke knowledge, 8-item modified Morisky scale, Salt intake.	At 1 year ± 1 month after enrollment: post-study questionnaires to the same random 400 participants: Stroke knowledge, 8-item modified Morisky scale, Salt intake.
	5-minute post-study diet screener for fruits/vegetables/grains via online or by phone for all participants	

4. SELECTION AND ENROLLMENT OF SUBJECTS

4.1 Identifying eligible patients for the interventions

Analysts from EBSA medical centers generate lists of patients with uncontrolled HTN on a weekly basis by identifying eligible adults with uncontrolled HTN from queries of the PMT database which is kept updated by QOS (as described above in the Preliminary data on PHASE). In order to increase the likelihood that the patients are able to participate in diet and lifestyle coaching program, we will include only those who are proficient in the English language and are not on home hospice. We will not study children or pregnant women because causes and treatments are distinct in these populations[71] and the studied intervention is not directed toward these etiologies. About 95% of KPNC members have a pharmacy benefit [Jim Chan, KP Pharmacy Outcomes Research Group, personal communication], which will allow tracking of prescriptions.

Table. Eligible patients for interventions

Inclusion criteria

- Kaiser Permanente member throughout the study period with pharmacy benefits.
- Age ≥18.
- In the HTN registry.
- Self-reported race of African American.
- Sufficient understanding of the English language.

Exclusion criteria

- Non-Kaiser Permanente member
- Age <18 or >85 years
- A race other than African American.
- Not included in HTN registry.
- Pregnant women.
- End-stage renal disease (ESRD)
- Dementia
- Members in hospice or on home hospice or have life expectancy of <6 months.
- Members in skilled nursing facility (SNF)
- Non-English speaking

5. STUDY INTERVENTIONS

5.1 Control Group

Usual Care. All patients whose PCPs are randomized to Control Group will continue their usual care for HTN management. Those with high cardiovascular risks would be in the PHASE population as described above in preliminary data section. These patients tend to be targeted for BP control by chronic care managers from each medical center based on their chronic conditions. Those with HTN but no other cardiovascular risks are typically managed by their PCPs or by a clinical pharmacist at a BP check visit. Patients with uncontrolled HTN are picked up by the weekly queries of the PMT database generated by medical center analysts. They would be contacted to come in for a **BP check visit which is free of charge**. How quickly their pharmacotherapy regimens get ramped up vary as our preliminary data indicate. Utilization of health

education services also varies depending on each PCP's recommendations and each patient's willingness to follow through.

At the BP check visit, a medical assistant (MA) checks the patient's BP, records measurements, asks patients about current medications and inquire about possible side effects from any medications. These are recorded in patient's electronic chart and then it is routed to a clinical pharmacist at the local center. The pharmacist will review current regimen and make recommendations regarding any changes/additions/deletions of any anti-hypertensive, and then routes the chart back to the MA. If the pharmacist is not available, the MA would receive a message of unavailability and would then route the chart to the PCP. The MA is responsible for communicating the pharmacist's or the PCP's recommendations to the patient and instructs the patient to go to the local pharmacy to pick up any new prescriptions. Most of the time, this can happen live. The other times, the MA would contact the patient later by phone to convey the recommendations. At the pharmacy, pharmacists are available to discuss the new prescription with the patient including potential side effects.

Those with uncontrolled hypertension who are randomized to the usual care arm will receive an initial letter describing the study, the potential benefits and risks associated with the study, and a reply postcard. The guidebook on "Caring for Your Family's Health: A Guide for African Americans" and DVD will be distributed free of charge to all arms. Developed in 2005 from a partnership between the American Academy of Family Physicians, Kaiser Permanente, and the Institute of Church Administration and Management, this guidebook stresses the importance of "knowing your numbers" for chronic illnesses such as HTN, diabetes, high cholesterol and heart disease. The video encourages African Americans to take charge of their health by knowing their family history and developing a positive relationship with their family physician.

Study staff will contact the patients after 2 week time if no reply postcard is received in order to discuss the study further, answer any question, and to obtain verbal consent for enrollment.

5.2 Diet & Lifestyle Coaching Intervention

In the treatment and prevention sciences, researchers have long recognized that public health and health behavior change interventions are best tailored to individuals' unique needs and concerns.[72, 73] In contrast to traditional one-size-fits-all treatment programs, programs designed with flexibility, tailored to each individual's needs, are generally more attractive to public policymakers because they focus more on those who need more attention and potentially can free up resources for more intensive treatment of the needy. These types of programs also hold the promise of reducing non-compliance by subjects due to over-treatment or under-treatment.[74] In addition, interventions tailored to overcome patient-specific barriers have been found to be the most promising approaches to enhance patient medication adherence.[75] Several recent randomized controlled trials have shown that nurse managers trained in behavioral approaches, specifically motivation-based, can improved medication adherence and improve BP control.[76, 77]

Enrollment. A list of all patients with uncontrolled HTN will be generated by an analyst from each medical center on a weekly basis. From these lists, a list will be generated with only African American patients with uncontrolled HTN belonging to PCPs randomized to the intervention. A letter will be sent to these patients welcoming them to the Kaiser HTN control program and informing them that they have been selected to receive individual coaching on diet and lifestyle by a trained wellness coach to help improve their HTN control. The letter will outline the intervention session schedule and the time commitment to participate. A reply postcard will be included. The patients will also have an opportunity to decline participation in the study, but give permission for the study investigators to review their medical records for information and health conditions relating to their blood pressure management. If the patient chooses not to opt out, a research assistant will contact the patient to schedule the sessions. The diet & lifestyle intervention will begin after enrollment and last for 12 months.

Group sessions. Participants will be asked to attend 2 group sessions at an EBSA medical center, at pre- and post-study. Attempts will be made to offer evening and weekend sessions to accommodate working

schedules. The pre-study group session will allow for introductions, completion of study assessments, an introduction to HTN, and distribution of culturally tailored education materials. The post-study group session will serve as a wrap-up to complete study assessments, provide feedback about the intervention and trial, and allow the research team to thank patients for participating.

Individual phone sessions. Participants will receive up to 16 telephone coaching sessions over a 1-year period with a Kaiser Permanente Wellness Coach: 4 sessions 1 week apart in Month 1; 4 additional sessions two weeks apart in Months 2 and 3; and then 4-8 additional sessions 4-6 weeks apart in Months 4-12. The first session will last about 30 minutes, the remaining sessions will last about 15 minutes each (Figure – study diagram).

Using educational materials, coaches will aim to increase patients' understanding of HTN, his/her own risks for future stroke and cardiovascular disease, and the current evidence about managing those risks (e.g., the importance of maintaining a low-salt diet and an active lifestyle). Based on Bandura's social cognitive theory (SCT)[78-80] and the Trans-theoretical model (TTM),[81] in addition to the Health Belief Model[45, 46] outlined above as the theoretical framework for the intervention, the protocol for individual sessions will follow a step-wise, phased approach to behavior change. This approach will focus on: a) helping participants establish a series of sequential, realistic, short-term goals for target behaviors; b) enabling participants to self-monitor performance on these goals in a way that builds self-efficacy, i.e., confidence in their ability to adhere to healthy behaviors across situations (previously shown to predict dietary[82] and PA goal achievement[83, 84]); and c) encouraging participants to define barriers to adherence and develop specific strategies for overcoming those barriers using a problem-solving approach.[85] Patients will be taught to self-monitor their dietary intake at least one day per week (Appendix K), as well as their physical activity and weight. Records can be faxed, emailed, or mailed to wellness coaches before each scheduled phone session. Coaches will use the records to provide feedback and collaboratively set goals tailored to patients' cultural, economic, and personal preferences, as well as their stage of change (i.e., readiness to adopt lifestyle changes). Coaches will assist participants in identifying social support for healthy nutrition[86] and physical activity[87] behaviors from family, friends, and peers. At the conclusion of each session, participants and coaches will identify action plans to accomplish goals and overcome barriers, and schedule the next session. Subsequent sessions will focus on assessing adherence, reviewing progress on previously set goals, and problem solving barriers as appropriate.

Coaches will employ motivational interviewing (MI) counseling techniques. MI promotes a patient's internal motivation to make and sustain health behavior changes, fostering autonomous motivation and self-efficacy to successfully execute self-care tasks.[88, 89] MI is consistent with key elements of the Health Belief Model (see section 11.1.4), including helping patients identify pros (benefits) and cons (barriers) of behavior change, and identifying facilitators to carrying out health recommendations. Consistent with the motivational interviewing framework, coaches will adopt a nonjudgmental, empathetic, and encouraging stance that supports an environment of collaboration, mutual respect, and trust.

Our coaches will explore barriers to adherence to recommended therapy and develop short-term action plans with patients. The factual and education materials on HTN and recommendations for management used by the wellness coach during these phone sessions are based on KPNC health education materials included in the Appendixes. The goal is to increase the patient's understanding of HTN, his/her own risks for future stroke and cardiovascular disease, what the current evidence says about managing those risks with regard to maintaining a low-salt diet and an active lifestyle, and to determine the patient's barriers to the treatment plan in order to provide support and motivate him/her to adhere to a healthier lifestyle.

During each session, the coaches would work on developing rapport with the patient and establishing an environment of collaboration, mutual respect, and trust. In addition, the coaches would strive to be nonjudgmental, empathetic, and encouraging. Patient-specific barriers to therapy will be explored at each session. The discussion will include a comparison of patient's risk factor(s) with target goals. The coaches will help each patient explore a general target behavior (e.g. eat more fruits) and come up with a short-term action plan with the patient. At the end of each session, the coaches will summarize the action plan and next step and schedule the next follow-up phone session. Follow-up sessions focus on assessing adherence, review of progress on prior action plans and additional action planning as appropriate.

Education materials. Participants will receive multimedia education materials addressing chronic disease management, BP control, and dietary recommendations. The guidebook on “Caring for Your Family’s Health: A Guide for African Americans” and DVD will be distributed free of charge to participants in the intervention arms (Appendix I). Developed in 2005 from a partnership between the American Academy of Family Physicians, Kaiser Permanente, and the Institute of Church Administration and Management, this guidebook stresses the importance of “knowing your numbers” for chronic illnesses such as HTN, diabetes, high cholesterol and heart disease. The video encourages African Americans to take charge of their health by knowing their family history and developing a positive relationship with their family physician. Wellness coaches will use the guidebook and DVD as a tool to deliver important health information and help African Americans to know their numbers better. For coaching on lifestyle and how to lower one’s BP, education materials will be taken from the guidebook & DVD (Appendix I) and the KPNC handout on HTN management (Appendix D). For coaching on diet, wellness coaches will utilize DASH diet handouts which include healthy examples from each food group as well as meal samples for 3 days (Appendix E).

Targets for the intervention. Participants will be encouraged to adhere to anti-hypertensive prescriptions, engage in smoking cessation, and maintain a healthy weight. Dietary targets will be based on the DASH diet, which advises 6-8 servings per day of whole grains, 4-5 servings per day of vegetables, 4-5 servings per day of fruits, low-fat/fat-free dairy products, and lower sodium intake of 2400mg per day. Participants will be encouraged to do regular physical activity for 30-60 minutes most days of the week in a step-wise fashion. For example, participants will first be encouraged to do something active 3-4 days per week; at subsequent sessions, the duration and frequency of activity recommended will be increased to meet the behavioral target. The participation target for the intervention is 50% of participants to complete 6 or more lifestyle sessions.

5.3 Enhanced BP Protocol Intervention

One of the key obstacles to achieving HTN control in African Americans has been identified as the failure of medical providers to treat high BP early and to continue to treat it persistently in order to maintain appropriate BP goals.[53] With that in mind, we have developed an intervention focusing on quickly identifying African Americans with uncontrolled HTN and targeting them early and aggressively and persistently with pharmacologic agents that have been effective in reducing BP in blacks. We expand upon the current infrastructure of BP check visits and utilization of clinical pharmacists to make quick modifications as necessary to a patient’s anti-hypertensive regimen without additional burden to the PCPs’ busy clinic schedule.

Enrollment. A list of all patients with uncontrolled HTN will be generated by an analyst from each medical center on a weekly basis from the PMT database. From these lists, a list will be generated with only African American patients with uncontrolled HTN belonging to PCPs randomized to this intervention arm. A letter will be sent to these patients to welcome them to the HTN control program in Kaiser and to emphasize the importance of prompt follow-ups for BP check visits should they become necessary when BP is uncontrolled. In addition, the guidebook on “Caring for Your Family’s Health: A Guide for African Americans” and DVD will be distributed free of charge to all arms. The initial letter will outline the intervention and the time commitment to participate. A reply postcard will be included. The patients will also have an opportunity to decline participation in the study, but give permission for the study investigators to review their medical records for information and health conditions relating to their blood pressure management. Study staff will contact the patients in 1 week time if no post card is received to discuss the study further, answer any questions and to obtain verbal consent for enrollment.

BP check visits. The weekly list of African American patients with uncontrolled HTN belonging to PCPs randomized to this intervention arm will be sent to our Research Coordinator and to the study clinical pharmacist. The Research Coordinator is responsible for contacting each patient and scheduling a BP check visit within 2-3 weeks. **Same as for usual care, these BP check visits are free of charge for all members.** A research assistant will help with reminder phone calls and secure messages several days before each scheduled appointment. BP check visits are available every day at all EBSA medical centers. A BP check clinic is available as part of urgent care for walk-ins on the weekends at Oakland Medical Center. The Research Coordinator will send a weekly list of scheduled patients to the MA group at each primary care clinic

station and also to the study clinical pharmacist. Every attempt will be made to reschedule promptly should a patient unable to make an appointment.

At the visit, a MA checks the patient's BP, record measurements, ask patients about current medications and inquire about possible side effects from any medications. These are recorded in patient's electronic chart and then it is routed to the Research Coordinator and the study clinical pharmacist. The pharmacist will review current regimen and make recommendations regarding any changes/additions/deletions of any anti-hypertensive, and then routes the chart back to the MA. The MA is responsible for communicating the pharmacist's recommendations to the patient and instructs the patient to go to the local pharmacy to pick up any new prescriptions. This will occur live whenever possible. At the pharmacy, local pharmacists are available to discuss the new prescription with the patient including potential side effects. For weekend visits, the patient may have to wait until the next normal business day to receive the recommendations from the study clinical pharmacist. Any dangerously high BP recorded would prompt a referral to the Emergency Department for further management.

The BP check visits would essentially be run very similarly to what is currently taking place for usual care, except for several important differences: 1) the MA will route the chart to our Research Coordinator and study clinical pharmacist who will handle the BP regimen modifications as needed; 2) our Research Coordinator and study clinical pharmacist will be trained in culturally tailoring care for African Americans (as detailed below); 3) our clinical study pharmacist will follow the current BP protocol but will pay extra attention to ensure that medications such as thiazide, calcium channel blockers, and spironolactone are included and maximized in the BP control regimen as appropriate for African Americans; and 4) our research staff will handle the scheduling of follow-up BP check visits as necessary and provide encouragement and reminders to help patients adhere to these visits.

Measuring adherence to intervention. The EMR will be used to gather information on adherence to filling of prescribed anti-hypertensives, medication types and dosages, and attendance at scheduled BP check visits. Basic labs including serum creatinine and sodium levels are easily obtainable through EMR. Data will also be available from a random subset of patients on salt intake and 24-hour urine sodium (see below). Participation target of the intervention is that 50% of the enhanced monitoring participants with persistent high blood pressure readings during intervention will have a counseling session with either an RN or a pharmacist.

6. CLINICAL AND LABORATORY EVALUATIONS

6.1 Schedule of activities and assessments.

	Screening	Randomization	Baseline/pre-intervention	During intervention	Post-intervention
Eligibility	X				
Welcome letter		X			
Stroke knowledge survey			X		X
Modified Morisky Scale			X		X
Salt intake questionnaire			X		X
24-hr urine sodium & creatinine			X	X (diet & lifestyle group only)	X
DASH diet tracker				X	
Fruits & veggies screener			X		X

6.2 Timing of Evaluations

Diet and Lifestyle Coaching Intervention. Participants will receive up to 16 telephone coaching sessions over a 1-year period with a Kaiser Permanente Wellness Coach: 4 sessions 1 week apart in Month 1; 4 additional sessions two weeks apart in Months 2 and 3; and then 4-8 additional sessions 4-6 weeks apart in Months 4-12. The first session will last about 30 minutes, the remaining sessions will last about 15 minutes each.

Enhanced BP Protocol Intervention. BP check visits will be scheduled for approximately 2 weeks from the last uncontrolled BP documented.

The following visit definition and windows will be used for follow-up assessments:

**Assessment/Visit**	**Window**	**Definition**
Randomization		Study Day 0
2 weeks	5 days	Day 9-19, inclusive
1 month	7 days	Day 23-37, inclusive
3 month	7 days	Day 83-97, inclusive
12 Month	30 days	Day 330-390, inclusive

Note: 1 month = 30 days

6.3 Recruitment and Consent

This is a quality improvement effort focusing on African Americans with uncontrolled BP in the KPNC East Bay Service Area. All PCPs from the 4 sites in EBSA will be randomized to the 3 arms. All PCPs randomized to the interventions will receive notification of the study and will be informed that all their African American patients with uncontrolled HTN will be treated the same way. Patients will be identified by analyst at each site from the HTN registry as having uncontrolled BP. Letters will be sent to welcome all patients to the KPNC blood pressure control program. The letter will outline the study and the time commitment to participate. An opt-out postcard will be included. The patients in the diet & lifestyle and in the enhanced protocol arms will also have an opportunity to decline participation in the study, but give permission for the study investigators to review their medical records for information and health conditions relating to their blood pressure management. For the diet & lifestyle intervention group, the letter will also inform the patients of their eligibility to receive the wellness coaching for free of charge to help them control their BP better. For the enhanced BP protocol intervention group, the letter will emphasize the importance of attending all BP check visits promptly as scheduled if indicated for their BP control. If the patients do not choose to opt out of the study, then the research staff and coaches will contact the patients for enrollment.

The trial carries minimal risks to the participants. Our local IRB has approved waiver of informed consent from our local IRB for usual care arm but informed consent would be obtained for enhanced and lifestyle arm.

6.4 Assessments

Diet and Lifestyle Coaching Intervention

Treatment receipt by participants (their engagement with and comprehension of intervention content) will be enhanced through the interactive nature of coaching sessions. Attendance at sessions will be tracked.

Treatment enactment by participants (their adherence to intervention behaviors) will be assessed by coaches' review of dietary self-monitoring records, as well as staff review of the EMR. The EMR will be used to gather information on adherence to filling of anti-hypertensive prescriptions, smoking status, weight management (i.e., BMI), and BP measurements. Results from the 24-hour urine sodium checks in a random subset of patients will also be used to correlate with information provided on a salt intake questionnaire (below). Additionally, diet will be assessed in all participants in this intervention group using an adaptation of the fruit /vegetable/fiber dietary screener (shown below) that estimates fruit and vegetable, and fiber intake developed by Block et al.[90, 91] Participants will be asked to recall frequency of intake of 7 items to estimate fruit and vegetables and 3 items to estimate fiber intake during the past 3 months. The questionnaire takes approximately 5 minutes to complete and can be completed either online or by phone, and will be done both

before pre- and post- intervention at the 1 year assessment. The screener has the ability to rank individuals for fruit and vegetable intake and may be analyzed using prediction equations to generate point estimates of total fruit/vegetable servings, Vitamin C (mg), magnesium (mg), potassium (mg), and dietary fiber (mg). The questionnaire has demonstrated good validity when compared to estimates from a full length food frequency questionnaire (spearman correlation coefficients: 0.71 fruit and vegetable servings, .0.62 dietary fiber).[91]

Web-based screener for fruits, vegetables and grains from nutritionquest.com

Fruits, Vegetables, and Grains	Less than 1/WEEK	Once a WEEK	2-3 times a WEEK	4-6 times a WEEK	Once a DAY	2+ a DAY
Submit Questionnaire Clear Values						
Fruit juice, like orange, apple, grape, fresh, frozen or canned. (Not sodas or other drinks)	○	○	○	○	○	○
How often do you eat any fruit, fresh or canned (not counting juice?)	○	○	○	○	○	○
Vegetable juice, like tomato juice, V-8, carrot	○	○	○	○	○	○
Green salad	○	○	○	○	○	○
Potatoes, any kind, including baked, mashed or french fried	○	○	○	○	○	○
Vegetable soup, or stew with vegetables	○	○	○	○	○	○
Any other vegetables, including string beans, peas, corn, broccoli or any other kind	○	○	○	○	○	○
Fiber cereals like Raisin Bran, Shredded Wheat or Fruit-n-Fiber	○	○	○	○	○	○
Beans such as baked beans, pinto, kidney, or lentils (not green beans)	○	○	○	○	○	○
Dark bread such as whole wheat or rye	○	○	○	○	○	○

Enhanced BP Protocol Intervention

The EMR will be used to gather information on adherence to filling of prescribed anti-hypertensives, medication types and dosages, and attendance at scheduled BP check visits. Basic labs including serum creatinine and sodium levels are easily obtainable through EMR. Data will also be available from a random subset of patients on salt intake and 24-hour urine sodium (see below).

Surveys and Sodium Tests

Surveys. Surveys on stroke knowledge and risk factors (Appendix F), 6-item Modified Morisky Scale (Appendix G), and salt intake (Appendix H) will be sent out at pre-study (baseline) and post-study (1 year post-enrollment) to a random sample of 400 African American patients with uncontrolled HTN from each arm of the trial.

24-hour urine sodium test. A random sample of 100 African Americans with uncontrolled HTN from each arm of the trial will receive a kit to collect 24-hour urine for sodium and creatinine tests. The kits will be mailed to those in the Control Group and handed out to those in the diet & lifestyle intervention at their group sessions at pre- and post-study, and will be collected at several drop-off locations in the East Bay upon completion. The participants can also schedule a drop off at the DOR or at a local Kaiser lab.

6.5 Definitions of Evaluations

Hypertension

We will be identifying African American patients with uncontrolled HTN from the KPNC HTN Registry. Analysts from the 4 medical centers in the study will generate a weekly list of patients with uncontrolled HTN using the Patient Management Tool (PMT) database which is kept up-to-date by the Department of Quality and Operations Services.

HNT Registry Criteria

- 2 or more primary care HTN diagnosis
- 1 or more primary care HTN diagnosis and 1 or more hospitalizations with a primary or secondary HTN diagnosis
- 1 or more primary care HTN diagnosis and 1 or more filled prescriptions for HTN medication within the last 6 months
- 1 or more primary care HTN diagnosis AND (1 or more stroke-related hospitalizations OR history of CAD, heart failure of diabetes)

Hypertension Control. Defined as having a BP <140/90 mm Hg.
Sustained BP control. Defined as having a BP <140/90 for at least 75% of the time in the study.

Definitions of Clinical Outcomes

- **Ischemic stroke:** An acute focal infarction of the brain or retina associated with neurological symptoms persisting more than 24 hours or of lesser duration but with death or documentation of acute brain infarction. Criteria: (1) rapid onset of a focal neurological deficit that lasts $\geq$24 hours and is not attributable to a nonischemic etiology (not associated with brain infection, trauma, or tumor, seizure, severe metabolic disease, or degenerative neurological disease); or, (2) rapid onset of a focal neurological deficit with associated acute ischemic changes in the brain seen on an imaging study.
- **TIA (as outcome):** A neurological deficit of sudden onset, resolving completely within 24 hours attributed to focal brain ischemia without evidence of associated acute focal infarction of the brain. Criteria: (1) rapid onset of a focal neurological deficit that lasts <24 hours and is not attributable to a nonischemic etiology (not associated with brain infection, trauma, or tumor, seizure, severe metabolic disease, or degenerative neurological disease); AND, (2) absence of lesions on acute brain imaging that are consistent with acute ischemia and could account for symptoms.
- **Hemorrhagic stroke:** An acute extravasation of blood into the brain parenchyma or subarachnoid space with associated symptoms. Criteria: (1) Evidence of hemorrhage in the brain parenchyma or subarachnoid space demonstrated by head imaging, surgery, or autopsy; AND (2) focal neurological deficit or other symptoms (eg, headache, meningismus) attributable to the hemorrhage.
- **Myocardial infarction:** Evidence of myocardial injury attributable to ischemia from coronary artery disease. Criteria: The diagnosis of MI will be based on the universal definition of myocardial infarction.[92] an algorithm developed for the HERS study that takes into account 3 categories of clinical information from the acute event: ischemic symptoms, ECG abnormalities, and elevated cardiac enzyme levels.[93, 94] The diagnosis will also be made if there was evidence of acute MI at autopsy
- **Major systemic hemorrhage:** Extracranial extravasation of blood, which is life-threatening or requires transfusion.[95] Criteria: demonstrated extravasation of blood associated with a reduction in hematocrit of at least 5%, requiring transfusion or resulting in hospitalization or death.
- **Other treatment-related complication (serious or non-serious):** Alteration in liver, renal, or hematological function or an allergic response that could be due to study medications. Criteria:

Systemic dysfunction that is considered by the treating physician possibly related to study medications. Serious complications are those that require hospitalization or medication discontinuation; others are non-serious.

- **Vascular death:** Death due to stroke (ischemic or hemorrhagic), systemic hemorrhage, myocardial infarction, congestive heart failure, pulmonary embolism, sudden death, or arrhythmia. Criteria: Death not clearly due to a nonvascular etiology.[95]
- All strokes will be adjudicated by 2 independent neurologists and disagreements will be settled by a third neurologist.

6.6 Medical History

Patient medical history including co-morbidities, surgical history, social history (smoking status), imaging studies, procedures, laboratory results, and all medications for other conditions is all available in our electronic medical record system.

6.7 Laboratory Evaluations

A random sample of 100 African Americans with uncontrolled HTN from each arm of the trial will receive a kit to collect 24-hour urine for sodium and creatinine tests. This will be done pre- and post-study for the control group and the enhanced BP protocol group. The diet & lifestyle coaching group will do this test pre-study, mid-study and post-study.

7. MANAGEMENT OF ADVERSE EXPERIENCES

The diet and lifestyle intervention is a wellness coaching program that will provide patients with information on how to live a healthy lifestyle including smoking cessation and being active, and eating a low-salt diet using guidelines for the DASH diet. The DASH diet which is rich in fruits and vegetables coupled with lower sodium intake has been shown in clinical trials to benefit African Americans the most in HTN control without any significant risks. Therefore we would anticipate that this wellness coaching intervention carries minimal risks to the participants and the potential benefits of HTN control would outweigh any minimal risks.

The enhanced BP protocol intervention uses the same BP protocol that is currently used in clinical care. Our clinical pharmacist will make recommendations on anti-hypertensives using the same list of medications and same dosages currently listed on the BP protocol. The differences from usual care are that the patients in this arm will be scheduled for frequent BP check visits as necessary to get their BP under control, and that the study clinical pharmacist will maximize the use of thiazide, calcium channel blocker, and spironolactone as needed to control BP. There is a potential for an increase usage of spironolactone in resistant HTN as it is currently much underutilized. Because spironolactone reduces the body's production of testosterone and also blocks its testosterone receptors, in men it can cause gynecomastia. A potential more serious side effect of spironolactone is hyperkalemia which can be monitored by blood works. Self-reported side effects are recorded during BP check visits. In addition, we will monitor for potential side effects recorded at other clinic visits and lab results by searching EMR.

8. CRITERIA FOR INTERVENTION DISCONTINUATION

An intervention would be discontinued if a subject has a positive serum pregnancy test or expresses a desire to become pregnant. Her HTN management would then be handled by her PCP and her OB physician as appropriate.

Subjects will be followed according to the study procedures as specified in this protocol up to the scheduled date of study completion. All subjects who had to discontinue an intervention will be followed per protocol to 12 months from randomization, or to death, whichever comes first. For subjects considered lost to follow-up, data must be collected up to the last visit performed. The Investigator should make every effort to contact the subject and to identify the reason why he/she failed to attend the scheduled visit or coaching

session and to determine his/her health status at 12 months by telephone contact with the subject or the subject's alternative contacts with last known recorded information from EMR.

9. STATISTICAL CONSIDERATIONS

9.1 General Design Issues

This is a pragmatic cluster randomized controlled comparative effectiveness trial with randomization at the primary care provider (PCP) level. The PCPs along with their patient panels will be randomized to one of 3 arms: 1) usual care; 2) diet and lifestyle coaching program; or 3) enhanced BP management with pharmacotherapy. The interventions will focus on African Americans with uncontrolled HTN (Figure– study flow diagram). We will identify eligible patients with uncontrolled HTN (defined as BP <140/90) using KPNC electronic databases and the HTN registry. In the two intervention arms, we will have an option for the patients to decline participation in the study, but give the study investigators permission to review their medical records for information and health conditions (such as stroke and cardiovascular disease) relating to their blood pressure management. Those who are randomized to the diet and lifestyle coaching intervention and opt to be in the study will be contacted by a trained wellness coach at regular intervals via telephone sessions as well as attending group education sessions (Figure – study flow diagram). Those who are randomized to the intensified BP management with pharmacotherapy arm will be contacted by a research coordinator to facilitate their BP check visits as needed and their medication regimens will be reviewed and modified as necessary by a clinical pharmacist. All research staff and providers will be trained in cultural competency and tailoring interventions for African Americans. Both interventions will be implemented for a one-year duration post-enrollment.

<u>The primary goal is to reduce HTN control disparity between blacks and whites by 4% at the end of 1 year.</u> The primary outcome will be the proportion of African Americans with HTN who have their blood pressure under control as assessed at the end of 1 year. Data for BP and clinical outcomes will continue to be collected for 36 months post-enrollment for secondary outcomes which will include BP control rates at 2 and 3-year post-enrollment, stroke/vascular death/myocardial infarction risk, and time stayed in BP control. Data will be analyzed by intention-to-treat. Demographics, adherence to prescriptions, socioeconomic and behavioral factors that may be related to success of the interventions will be examined.

Randomization

There are 194 PCPs in the EBSA with 191 providers having African Americans in their patient panels. The unit of randomization will be the primary care providers in the EBSA (N=191) who have black patients in their panels. The other 3 PCPs can be randomized into the study at a later date if and when they add African American patients to their panels. All PCPs will be randomized to one of the 3 arms. To the extent possible, we will strive to have an approximately equal number of African Americans with HTN in each arm of the trial (approximately 1896 with uncontrolled HTN expected per group). This will be accomplished by using stratified randomization with blocking in units of three. The strata will be 3 categories determined by the number of black patients with hypertension in each clinician's panel.

With the enhanced BP protocol intervention, because it is a quality improvement effort, randomizing at the provider level will allow all African American patients with HTN to be targeted for the intervention should their BP ever become uncontrolled. The IRB required that patients assigned to the enhanced BP protocol intervention must give informed consent. Being able to include all African Americans in this intervention will increase the generalizability of the study findings.

With the diet and lifestyle coaching intervention, randomizing at the provider level will also allow us to invite all African American patients with uncontrolled HTN to participate in this intervention. Even though the materials used in developing this intervention are available within KPNC, a formal coaching program for diet

and lifestyle effects on HTN is currently non-existent. We will send out letters to inform eligible patients of this on-going improvement project. The patients will have the option of opting out of the study by returning a stamped envelope to us. If the patients choose not to opt out, then a research staff will contact them to discuss enrollment as scheduled (Figure – study diagram, above).

As detailed in the Statistical Analysis Section we anticipate enrollment of approximately 1896 blacks with uncontrolled HTN to each arm of the study spread out over a 2-year period. The randomization process of all PCPs will be carried out by a programmer at the KPNC DOR offices (Oakland, CA).

9.2.1 Primary outcome

The primary outcome measure is the proportion of African American patients with HTN who achieved BP control at 1 year post-study enrollment. Controlled BP control is defined as having SBP <140 and DBP <90.

9.2.2 Secondary outcomes

The following secondary outcomes will also be evaluated using a similar analysis plan as outlined above:

- Combined outcome of stroke occurrence, vascular death, or myocardial infarction.
- Readmission with stroke (ICD-9 430-439).
- All death.
- Myocardial infarction.
- Time to first BP control.
- Proportion of time with BP under control.
- Use of spironolactone.
- Mean change in systolic and diastolic BP
- Per protocol BP control at 1 year post-study enrollment

9.3 Sample Size and Accrual

Currently, 191 clinicians have 15,609 adult African American patients with hypertension, 3258 (20.9%) of which have uncontrolled HTN. The 191 clinicians will be randomized to the three study groups, stratifying on panel-size, resulting in approximately 64 clinicians (clusters) in two of the groups and 63 in the other; and approximately 1085 patients per arm. Using data from the past year, we estimate that following: 1) 1307 of our original patients with HTN will change from being in control to uncontrolled over the course of 1 year; and 2) there will be 1125 newly diagnosed African American patients with hypertension that is out of control in a 1 year period. Therefore, we estimate that we will include approximately 1896 African American patients with uncontrolled HTN in each arm of the study over the one year recruitment period. Given this fixed number of patients, we will calculate the power we will have to detect an overall reduction of 4% in HTN control disparity between black and white patients. In addition, we estimated that the rate of uncontrolled HTN is decreasing by approximately 1% per year among whites without the effect of our proposed interventions. We would therefore need a reduction of 5% in the proportion of African American patients with hypertension who are out of control in order to say that there is a 4% reduction in disparity due to the treatment.

To estimate the between cluster coefficient of variation, we used the current information on the adult African American patients with HTN in the EBSA within each of the 191 panels. We took 100 random samples of clusters from the 191, each with approximately one third of the 191 clusters. For each sample, we calculated a between cluster coefficient of variation (cv), and then calculated the mean of these 100 cvs, resulting in an estimate of 0.0806. Assuming a baseline proportion of uncontrolled hypertension in blacks to be 19.9%, and that in whites to be 14.7%, a background reduction of 1% per year, and a Type I error rate of 0.025, we would have >99% power to detect 4% reduction in proportions due just to the treatment effect.

These calculations have assumed that there is no drop-out or loss to follow-up. In this study design, drop-out or loss to follow up can occur either at the cluster level (the clinician) or at the individual item level (the

patient). If a clinician left or transferred to a different facility, their patients would be referred to one of the other clinicians in their facility. The patients could continue to receive the intervention assigned to their original clinician since the interventions are not administered by the clinician. As far as a patient being lost to follow up, very few KPNC members leave the health plan over the course of one year, especially those with chronic conditions such as HTN. As long as they are still in the health plan, even if they decide to stop participating in the study, we would still be able to capture their BP measurements and cardiovascular outcomes using the administrative databases. The expected sample size is very robust to loss to follow up. Although we expect to have 1896 patients in each study arm, looking at the above power calculations a different way, we would have 80% power to detect an overall treatment effect of 4% even if we only had 3 subjects per clinician cluster or approximately 180 people in each study arm.

We are particularly interested in HTN control in patients under 45 years old. These patients made up approximately 15% of the patients with uncontrolled HTN. We would therefore have approximately 284 of these patients in each treatment arm. Using the same calculations as above for the whole group, in this subgroup we would have >99% power to detect an overall effect of at least 3%, and a 96% power to detect an overall effect of 2%.

For the dichotomous variables in the secondary aim, we will have the same power to detect the differences explained in the above table for the primary outcome. For the proportion of time the BP is in control over each follow-up period, we will have more than 99% power to detect a difference in the means of treatment and control group of at least 0.2 standard deviations.

9.4 Data Monitoring

The NINDS Guidelines on Data and Safety Monitoring generally require that a NINDS-appointed Data and Safety Monitoring Board monitor Phase III clinical trials. Describe the interim monitoring plan, including the schedule of interim analyses and guidelines for stopping the study for reasons of efficacy, safety, futility, or poor study performance (e.g., slow accrual, high losses-to-followup, poor quality control). Note that interim monitoring (for safety and study performance, at least) must be done at least annually following the randomization of the first subject. If the study includes stratification factors, indicate whether there are separate monitoring considerations for each stratum.

9.5 Data Analyses

All analyses will be done using the intent-to-treat method, analyzing every patient within the study group to which their clinician was randomized, regardless of whether or not the patient received their assigned treatment. This preserves the balance of the distribution of covariates among the three groups. Assumptions of all tests and models will be evaluated, and alternative methods used as needed. All analyses will be done using SAS version 9.3.

<u>Primary Aim:</u>

The primary research question of this study is to determine “whether a primary prevention intervention of either diet and lifestyle coaching, or an enhanced pharmacotherapy protocol is more effective than usual care in improving rates of HTN control in blacks and thereby reducing disparities between African Americans and whites.” The primary aim is to assess whether “by implementing either intervention, we will reduce the disparity in HTN control rates between blacks and whites by 4% at 1 year post-study enrollment.”

To address this aim, we will conduct a cluster-randomized clinical trial with three study arms: diet & lifestyle coaching intervention, enhanced pharmacotherapy intervention, and usual care. The unit of randomization will be the clinician, with all adult African American members of each clinician’s panel with hypertension being the members of that clinician’s cluster. Currently there are 191 clinicians (out of a total of 194) in the East Bay area of KPNC who have adult African American patients with hypertension in their panel, with an average of 81.7 patients per panel. Cluster sizes range from 1 to 419 patients per panel. Given the

wide range of these panel sizes, the randomization will be done within strata defined by the size of the clinician's panel of African American patients with hypertension: 41 panels with 1-10 such patients, 34 with 11-50 patients, 57 with 51-100 patients, and 59 with more than 100 patients. The randomization will also be done using block sizes of 3 to insure that a similar number of clusters will be in each treatment arm.

In each of the analyses explained below, we will separately compare the patients in each treatment arm to those in the control arm. To conservatively account for multiple comparisons error inflation, we will use a Type I error rate of 0.025.

The analysis will begin with a comparison of the baseline characteristics of the patients within each treatment arm to those in the control arm, in particular their age, gender, socio-economic status (marital status, household income by geocoding, living arrangement), number of anti-hypertensive drugs taking, types of anti-hypertensive drugs taking, 24-hour urine sodium, whether or not they have pharmacy coverage, and whether or not they are in the PHASE population. Categorical variables will be summarized using frequencies and proportions, and will be compared using chi-squared tests. Continuous variables will be summarized using means and standard deviations, or medians and interquartile ranges depending on the distributions of the variables. Comparisons between the control and each treatment group will be done using either two-sample t-tests or Wilcoxon rank sum tests, again depending on the distribution of each continuous variable. The primary outcome is the status of HTN control at 1 year after each patient is identified as having uncontrolled HTN. This will be compared between groups using a generalized linear mixed effects model (logit link) which will account for the within-cluster (clinician) correlation among patients. Clusters will be included in the model as a random effect. The primary independent variable in this model will be treatment group. We will also consider the inclusion of possible confounding variables, and in particular those variables found to be significantly imbalanced at baseline, by including each one in separate models with the treatment variable, and noting its impact on the treatment effect. Those variables which have a significant effect on the magnitude or precision of the treatment coefficient will be included in subsequent models. Results of these models will be summarized by odds ratios and their associated 95% confidence intervals.

All measures of adherence will be calculated and summarized for each treatment arm using means, medians, or frequencies as appropriate.

<u>Secondary aims:</u> 1) BP control at 2 and at 3 years post-enrollment; 2) a combined endpoint of stroke, major cardiovascular event, or death due to vascular causes; and 3) percentage of time BP stays under control at 1-year, 2-years, and 3-years post-enrollment.

The analysis for the first two endpoints will proceed in the same way as explained above for BP control at 1 year post-enrollment. The third outcome will also be modeled in the same way with the substitution of an identity link for the logit link.

10. DATA COLLECTION, SITE MONITORING, AND ADVERSE EXPERIENCE REPORTING

10.1 Records to be Kept

Aside from the surveys, diet screener and 24-hour urine sodium test, all other data on the subjects are kept as part of his/her EMR which is only accessible by authorized users with secured logins and passwords. The results of the surveys, diet screener and 24-hour urine test will be entered by a data entry staff into a secured study database that will be kept on a secured server at KPNC DOR. The server is accessible by our study programmer and biostatistician.

10.2 Role of Data Management

To protect privacy, only patients who have chosen to participate in the wellness coaching program will be approached by study investigators. The schedule of contacts and visits will be included in the information letter, so that those wishing to preserve privacy can exclude themselves from participation.

To protect confidentiality, the study database that contains unique identifiers will be maintained on a

single computer hard drive. An additional copy will be stored on CD. Only key study personnel will be allowed access to the database with unique identifiers. The working database will include a patient ID that will not be linked to unique identifiers.

10.3 Quality Assurance

Most of the data collected for this trial will come from KPNC EMR system. KPNC Department of Quality and Operations Services and the Informational Technology (IT) department oversee the EMR system.

For the diet and lifestyle coaching intervention, fidelity to protocol by the coaches will be assessed using standardized wellness coach training and evaluation of treatment delivery.

Wellness coach training. Wellness coaches will undergo training workshops on stroke knowledge including warning signs and symptoms and risk factors, as well as secondary prevention measures specific for HTN. Coaches will also undergo training in Motivational Interviewing (MI) and the specific modules and algorithms developed for this study. Several recent RCTs have shown that developing and retaining proficiency in MI requires specific training with follow up reinforcement.[96, 97] Training and supervision in MI will be conducted by a Master Trainer from KPNC's Regional Health Education department, Mindy Boccio. Ms. Boccio has 5 years of experience as the leader of the KPNC MI-based "Brief Negotiation" program and has worked with Dr. Schmittdiel (co-investigator on the proposed study) on previous intervention studies within KPNC.[98] Coaches will participate in an initial 3-day MI skills workshop in Oakland, CA. Four to six weeks after initiation of the intervention period, coaches will receive 4 hours of 1:1 observation and coaching in his /her clinical setting. Ms. Boccio will observe/listen to in-person and telephone sessions and provide immediate feedback to the coaches for permitted participants. To maintain proficiency, coaches will then receive individual telephone supervision/booster sessions every 3 months for the duration of the intervention period, providing a forum for problem solving barriers to effective counseling, role-playing, content reinforcement and deepening understanding of MI.

During the finalization of the study protocol (see timeline), we will conduct a short lead-in phase of the study. Coaches will be able to pilot a one-time intervention session with a small random number of African Americans with HTN. At this time, we will also pilot the pre- and post-study stroke knowledge survey (Appendix F), the Morisky Scale (Appendix G), and the salt intake questionnaire (Appendix H). In addition, wellness coaches will be notified of any updates on guideline changes on HTN management during regular meetings throughout the study period. Given the short duration of the intervention phase, we do not anticipate there will be any changes in the guidelines for the HTN management.

Treatment delivery by wellness coaches will be standardized in length (60 minutes for group sessions, 30 minutes for the first individual session, 15 minutes for subsequent individual sessions); frequency (4 sessions 1 week apart in Month 1; 4 additional sessions two weeks apart in Months 2 and 3; and then 4-8 additional sessions 4-6 weeks apart in Months 4-12 duration, up to 16 telephone coaching sessions over a 1-year period); and content (through written protocols and scripts for coaches, which will be developed in consultation with Regional Health Education staff). Coaches' adherence to intervention protocols and the MI approach will be monitored through individual supervision and direct and indirect (audio recorded) observation. A trained coder will assess 10% of recordings using the Motivational Interviewing Treatment Integrity code (MITI), a tool to enhance post-training proficiency.

Research Staff Training. Same as for our wellness coaches, our Research Coordinator and clinical pharmacist will undergo training workshops on stroke knowledge including warning signs and symptoms and risk factors, as well as secondary prevention measures specific for HTN. In addition, to support effective adoption of the intervention's counseling approaches, they will also undergo training in MI technique (details included above under training for wellness coaches). Four to six weeks after initiation of the intervention period, coaches will receive 4 hours of 1:1 observation and coaching in his /her clinical setting. Ms. Boccio will observe/listen to either live or recorded telephone sessions and provide immediate feedback to the Research Nurse Coordinator for permitted participants. Cultural competency training will be required for all

interventionists on this study. More discussion of this training is detailed below. Our Research Coordinator and clinical pharmacist will also receive training in approaches to increase medication and treatment adherence.

10.4 Adverse Experience Reporting

The greatest risk to patient health is mainly possible side effects from a potential increase usage of spironolactone. Potential side effects will be monitored at BP check visits as well as searches in EMR on a monthly basis for mention of gynecomastia, and lab results for hyperkalemia. We have reviewed the NINDS guidelines on data and safety monitoring, and this trial carries minimal risks (http://www.ninds.nih.gov/research/clinical_research/policies/data_safety_monitoring.htm#2_4). Based on section 2.4 in the NINDS guidelines, the study may be adequately monitored by the study PI and his/her IRB. It is expected that the PI will be actively involved in reviewing the progress of each subject on study and will bring to the attention of the IRB adverse events and unexpected problems that may influence the IRB's decision to allow the trial to continue, in accordance with the IRB's policies. In addition, the PI is expected to notify the NINDS program director.

11. HUMAN SUBJECTS

11.1 Institutional Review Board (IRB)

This protocol and any subsequent modifications will be reviewed and approved by the IRB. The principal investigator will apply for a waiver of informed consent for this study. No study subjects may be recruited until documented IRB approval is obtained. The principal investigator is responsible for following the requirements of the local IRB on periodic reporting of the progress of the study, reporting of serious or unexpected adverse events, safety monitoring reports and termination of the study.

11.2 Subject Confidentiality

Subjects' medical records in KPNC are all electronic and can only be accessed by authorized users with their own identifications and passwords. All laboratory specimens, evaluation forms, reports, and other records that leave the site will be identified only by the Study Identification Number (SID) to maintain subject confidentiality. All records will be kept in a locked file cabinet. All computer entry and networking programs will be done using SIDs only. Clinical information will not be released without written permission of the subject, except as necessary for monitoring by IRB.

11.3 Study Modification/Discontinuation

The study may be modified or discontinued at any time by the IRB, the NINDS, the sponsor, the OHRP, the FDA, or other government agencies as part of their duties to en-sure that research subjects are protected.

12. PUBLICATION OF RESEARCH FINDINGS

Publication of the results of this trial will be governed by the policies and procedures developed by the Executive Committee. The trial results will be published as soon as possible after database lockdown.

This trial will produce detailed data on treatment effects, medical care, and outcomes in a cohort of approximately 5687 African Americans with uncontrolled hypertension. Three years after the primary publication associated with this work is submitted, a HIPAA-compliant de-identified version of the database will be made available publicly. Shake, Rattle & Roll biostatistician will be consulted to assure that it is impossible to uniquely identify any participant. This may mean removing or categorizing certain variables. A data use agreement will not be required for access to this dataset. Diskettes with the data in comma-delimited text format will be sent to parties that express interest, including a data dictionary in a text file.

13. REFERENCES

1. Roger VL, Go AS, Lloyd-Jones DM, Adams RJ, Berry JD, Brown TM, Carnethon MR, Dai S, de Simone G, Ford ES, Fox CS, Fullerton HJ, Gillespie C, Greenlund KJ, Hailpern SM, Heit JA, Ho PM, Howard VJ, Kissela BM, Kittner SJ, Lackland DT, Lichtman JH, Lisabeth LD, Makuc DM, Marcus GM, Marelli A, Matchar DB, McDermott MM, Meigs JB, Moy CS, Mozaffarian D, Mussolino ME, Nichol G, Paynter NP, Rosamond WD, Sorlie PD, Stafford RS, Turan TN, Turner MB, Wong ND, Wylie-Rosett J. Heart disease and stroke statistics--2011 update: A report from the american heart association. *Circulation*.123:e18-e209
2. Lloyd-Jones D, Adams R, Carnethon M, De Simone G, Ferguson TB, Flegal K, Ford E, Furie K, Go A, Greenlund K, Haase N, Hailpern S, Ho M, Howard V, Kissela B, Kittner S, Lackland D, Lisabeth L, Marelli A, McDermott M, Meigs J, Mozaffarian D, Nichol G, O'Donnell C, Roger V, Rosamond W, Sacco R, Sorlie P, Stafford R, Steinberger J, Thom T, Wasserthiel-Smoller S, Wong N, Wylie-Rosett J, Hong Y. Heart disease and stroke statistics--2009 update: A report from the american heart association statistics committee and stroke statistics subcommittee. *Circulation*. 2009;119:480-486
3. Williams GR. Incidence and characteristics of total stroke in the united states. *BMC Neurol*. 2001;1:2
4. Sacco RL, Shi T, Zamanillo MC, Kargman DE. Predictors of mortality and recurrence after hospitalized cerebral infarction in an urban community: The northern manhattan stroke study. *Neurology*. 1994;44:626-634
5. Hankey GJ, Jamrozik K, Broadhurst RJ, Forbes S, Burvill PW, Anderson CS, Stewart-Wynne EG. Long-term risk of first recurrent stroke in the perth community stroke study. *Stroke*. 1998;29:2491-2500
6. Burn J, Dennis MS, Bamford JM, Sandercock PA, Wade D, Warlow CP. Long-term risk of recurrent stroke after a first-ever stroke. The oxfordshire community stroke project. *Stroke*. 1994;25:333-337
7. Petty GW, Brown RDJ, Whisnant JP, Sicks JD, O'Fallon WM, Wiebers DO. Survival and recurrence after first cerebral infarction: A population-based study in rochester, minnesota, 1975 through 1989. *Neurology*. 1998;50:208-216
8. Sacco RL, Boden-Albala B, Gan R, Chen X, Kargman DE, Shea S, Paik MC, Hauser WA. Stroke incidence among white, black, and hispanic residents of an urban community: The northern manhattan stroke study. *Am J Epidemiol*. 1998;147:259-268
9. Broderick J, Brott T, Kothari R, Miller R, Khoury J, Pancioli A, Gebel J, Mills D, Minneci L, Shukla R. The greater cincinnati/northern kentucky stroke study: Preliminary first-ever and total incidence rates of stroke among blacks. *Stroke*. 1998;29:415-421
10. Howard G, Anderson R, Sorlie P, Andrews V, Backlund E, Burke GL. Ethnic differences in stroke mortality between non-hispanic whites, hispanic whites, and blacks. The national longitudinal mortality study. *Stroke*. 1994;25:2120-2125
11. Rosamond WD, Folsom AR, Chambless LE, Wang CH, McGovern PG, Howard G, Copper LS, Shahar E. Stroke incidence and survival among middle-aged adults: 9-year follow-up of the atherosclerosis risk in communities (aric) cohort. *Stroke*. 1999;30:736-743
12. Sheinart KF, Tuhrim S, Horowitz DR, Weinberger J, Goldman M, Godbold JH. Stroke recurrence is more frequent in blacks and hispanics. *Neuroepidemiology*. 1998;17:188-198
13. Kennedy BS. Does race predict stroke readmission? An analysis using the truncated negative binomial model. *J Natl Med Assoc*. 2005;97:699-713
14. Rosamond W, Flegal K, Friday G, Furie K, Go A, Greenlund K, Haase N, Ho M, Howard V, Kissela B, Kittner S, Lloyd-Jones D, McDermott M, Meigs J, Moy C, Nichol G, O'Donnell CJ, Roger V, Rumsfeld J, Sorlie P, Steinberger J, Thom T, Wasserthiel-Smoller S, Hong Y. Heart disease and stroke statistics--2007 update: A report from the american heart association statistics committee and stroke statistics subcommittee. *Circulation*. 2007;115:e69-171
15. Appel LJ, Moore TJ, Obarzanek E, Vollmer WM, Svetkey LP, Sacks FM, Bray GA, Vogt TM, Cutler JA, Windhauser MM, Lin PH, Karanja N. A clinical trial of the effects of dietary patterns on blood pressure. Dash collaborative research group. *N Engl J Med*. 1997;336:1117-1124

16. Hertz RP, Unger AN, Cornell JA, Saunders E. Racial disparities in hypertension prevalence, awareness, and management. *Arch Intern Med*. 2005;165:2098-2104
17. Howard G, Prineas R, Moy C, Cushman M, Kellum M, Temple E, Graham A, Howard V. Racial and geographic differences in awareness, treatment, and control of hypertension: The reasons for geographic and racial differences in stroke study. *Stroke*. 2006;37:1171-1178
18. Gorelick PB. Stroke prevention. An opportunity for efficient utilization of health care resources during the coming decade. *Stroke*. 1994;25:220-224
19. Collins R, Peto R, MacMahon S, Hebert P, Fiebach NH, Eberlein KA, Godwin J, Qizilbash N, Taylor JO, Hennekens CH. Blood pressure, stroke, and coronary heart disease. Part 2, short-term reductions in blood pressure: Overview of randomised drug trials in their epidemiological context. *Lancet*. 1990;335:827-838
20. Gueyffier F, Boissel JP, Boutitie F, Pocock S, Coope J, Cutler J, Ekbom T, Fagard R, Friedman L, Kerlikowske K, Perry M, Prineas R, Schron E. Effect of antihypertensive treatment in patients having already suffered from stroke. Gathering the evidence. The indana (individual data analysis of antihypertensive intervention trials) project collaborators. *Stroke*. 1997;28:2557-2562
21. Progress collaborative group. Randomised trial of a perindopril-based blood-pressure-lowering regimen among 6105 individuals with previous stroke or transient ischaemic attack. *Lancet*. 2001;358:1033-1041
22. Bosch J, Yusuf S, Pogue J, Sleight P, Lonn E, Rangoonwala B, Davies R, Ostergren J, Probstfield J. Use of ramipril in preventing stroke: Double blind randomised trial. *Bmj*. 2002;324:699-702
23. Sacco RL, Adams R, Albers G, Alberts MJ, Benavente O, Furie K, Goldstein LB, Gorelick P, Halperin J, Harbaugh R, Johnston SC, Katzan I, Kelly-Hayes M, Kenton EJ, Marks M, Schwamm LH, Tomsick T. Guidelines for prevention of stroke in patients with ischemic stroke or transient ischemic attack: A statement for healthcare professionals from the american heart association/american stroke association council on stroke: Co-sponsored by the council on cardiovascular radiology and intervention: The american academy of neurology affirms the value of this guideline. *Stroke*. 2006;37:577-617
24. Psaty BM, Smith NL, Siscovick DS, Koepsell TD, Weiss NS, Heckbert SR, Lemaitre RN, Wagner EH, Furberg CD. Health outcomes associated with antihypertensive therapies used as first-line agents. A systematic review and meta-analysis. *Jama*. 1997;277:739-745
25. Messerli FH, Grossman E, Goldbourt U. Are beta-blockers efficacious as first-line therapy for hypertension in the elderly? A systematic review. *Jama*. 1998;279:1903-1907
26. Hansson L, Lindholm LH, Niskanen L, Lanke J, Hedner T, Niklason A, Luomanmaki K, Dahlof B, de Faire U, Morlin C, Karlberg BE, Wester PO, Bjorck JE. Effect of angiotensin-converting-enzyme inhibition compared with conventional therapy on cardiovascular morbidity and mortality in hypertension: The captopril prevention project (cappp) randomised trial. *Lancet*. 1999;353:611-616
27. Staessen JA, Fagard R, Thijs L, Celis H, Arabidze GG, Birkenhager WH, Bulpitt CJ, de Leeuw PW, Dollery CT, Fletcher AE, Forette F, Leonetti G, Nachev C, O'Brien ET, Rosenfeld J, Rodicio JL, Tuomilehto J, Zanchetti A. Randomised double-blind comparison of placebo and active treatment for older patients with isolated systolic hypertension. The systolic hypertension in europe (syst-eur) trial investigators. *Lancet*. 1997;350:757-764
28. Major outcomes in high-risk hypertensive patients randomized to angiotensin-converting enzyme inhibitor or calcium channel blocker vs diuretic: The antihypertensive and lipid-lowering treatment to prevent heart attack trial (allhat). *Jama*. 2002;288:2981-2997
29. Dahlof B, Devereux RB, Kjeldsen SE. Cardiovascular morbidity and mortality in the losartan intervention for endpoint reduction in hypertension study (life): A randomised trial against atenolol. *Lancet*. 2002;359:995-1003
30. U.S. Department of health and human services. Healthy people 2020 summary of objectives: Heart disease and stroke. 2012
31. Racial/ethnic disparities in prevalence, treatment, and control of hypertension--united states, 1999-2002. *MMWR Morb Mortal Wkly Rep*. 2005;54:7-9
32. Centers for disease control and prevention. A closer look at african american men and high blood pressure control: A review of psychosocial factors and systems-level interventions. 2010
33. Bravata DM, Wells CK, Gulanski B, Kernan WN, Brass LM, Long J, Concato J. Racial disparities in stroke risk factors: The impact of socioeconomic status. *Stroke*. 2005;36:1507-1511

34. Moy E, Bartman BA, Weir MR. Access to hypertensive care. Effects of income, insurance, and source of care. *Arch Intern Med*. 1995;155:1497-1502
35. Douglas JG, Bakris GL, Epstein M, Ferdinand KC, Ferrario C, Flack JM, Jamerson KA, Jones WE, Haywood J, Maxey R, Ofili EO, Saunders E, Schiffrin EL, Sica DA, Sowers JR, Vidt DG. Management of high blood pressure in african americans: Consensus statement of the hypertension in african americans working group of the international society on hypertension in blacks. *Arch Intern Med*. 2003;163:525-541
36. Lukoschek P. African americans' beliefs and attitudes regarding hypertension and its treatment: A qualitative study. *J Health Care Poor Underserved*. 2003;14:566-587
37. Kotchen TA, McCarron DA. Dietary electrolytes and blood pressure: A statement for healthcare professionals from the american heart association nutrition committee. *Circulation*. 1998;98:613-617
38. Krauss RM, Eckel RH, Howard B, Appel LJ, Daniels SR, Deckelbaum RJ, Erdman JW, Jr., Kris-Etherton P, Goldberg IJ, Kotchen TA, Lichtenstein AH, Mitch WE, Mullis R, Robinson K, Wylie-Rosett J, St Jeor S, Suttie J, Tribble DL, Bazzarre TL. Aha dietary guidelines: Revision 2000: A statement for healthcare professionals from the nutrition committee of the american heart association. *Circulation*. 2000;102:2284-2299
39. Neaton JD, Grimm RH, Jr., Prineas RJ, Stamler J, Grandits GA, Elmer PJ, Cutler JA, Flack JM, Schoenberger JA, McDonald R, et al. Treatment of mild hypertension study. Final results. Treatment of mild hypertension study research group. *JAMA*. 1993;270:713-724
40. Weir MR, Hanes DS. Hypertension in african americans: A paradigm of metabolic disarray. *Semin Nephrol*. 1996;16:102-109
41. Weir MR. Salt intake and hypertensive renal injury in african-americans. A therapeutic perspective. *Am J Hypertens*. 1995;8:635-644
42. Morris RC, Jr., Sebastian A, Forman A, Tanaka M, Schmidlin O. Normotensive salt sensitivity: Effects of race and dietary potassium. *Hypertension*. 1999;33:18-23
43. Sacks FM, Svetkey LP, Vollmer WM, Appel LJ, Bray GA, Harsha D, Obarzanek E, Conlin PR, Miller ER, 3rd, Simons-Morton DG, Karanja N, Lin PH. Effects on blood pressure of reduced dietary sodium and the dietary approaches to stop hypertension (dash) diet. Dash-sodium collaborative research group. *N Engl J Med*. 2001;344:3-10
44. Abraham S, Sheeran P. The health belief model. In: Conner M, Norman P, eds. *Predicting health behaviour*. Buckingham: Open University Press; 2005:23-61.
45. Becker MH. *The health belief model and personal health behavior*. New Jersey: Charles B. Slack, Inc.; 1974.
46. Rosenstock IM, Strecher VJ, Becker MH. Social learning theory and the health belief model. *Health Educ Q*. 1988;15:175-183
47. Glanz K, Rimer BK, Lewis FM. *Health behavior and health education. Theory, research and practice.* San Francisco: Wiley & Sons; 2002.
48. Stafford L, Jackson HJ, Berk M. Illness beliefs about heart disease and adherence to secondary prevention regimens. *Psychosom Med*. 2008;70:942-948
49. Ali NS. Prediction of coronary heart disease preventive behaviors in women: A test of the health belief model. *Women Health*. 2002;35:83-96
50. Sullivan K, Waugh D. Toward the development of the cerebrovascular attitudes and beliefs scale (cabs): A measure of stroke-related health beliefs. *Top Stroke Rehabil*. 2007;14:41-51
51. Sullivan KA, White KM, Young RM, Scott C. The cerebrovascular attitudes and beliefs scale (cabs-r): The factor structure and psychometric properties of a tool for assessing stroke-related health beliefs. *Int J Behav Med*. 2010;17:67-73
52. Sullivan KA, Katajamaki A. Stroke education: Promising effects on the health beliefs of those at risk. *Top Stroke Rehabil*. 2009;16:377-387
53. Oliveria SA, Lapuerta P, McCarthy BD, L'Italien GJ, Berlowitz DR, Asch SM. Physician-related barriers to the effective management of uncontrolled hypertension. *Arch Intern Med*. 2002;162:413-420
54. Richardson AD, Piepho RW. Effect of race on hypertension and antihypertensive therapy. *Int J Clin Pharmacol Ther*. 2000;38:75-79

55. Saunders E, Weir MR, Kong BW, Hollifield J, Gray J, Vertes V, Sowers JR, Zemel MB, Curry C, Schoenberger J, et al. A comparison of the efficacy and safety of a beta-blocker, a calcium channel blocker, and a converting enzyme inhibitor in hypertensive blacks. *Arch Intern Med*. 1990;150:1707-1713
56. Rahman M, Douglas JG, Wright JT, Jr. Pathophysiology and treatment implications of hypertension in the african-american population. *Endocrinol Metab Clin North Am*. 1997;26:125-144
57. Cushman WC, Reda DJ, Perry HM, Williams D, Abdellatif M, Materson BJ. Regional and racial differences in response to antihypertensive medication use in a randomized controlled trial of men with hypertension in the united states. Department of veterans affairs cooperative study group on antihypertensive agents. *Arch Intern Med*. 2000;160:825-831
58. Materson BJ, Reda DJ, Williams D. Lessons from combination therapy in veterans affairs studies. Department of veterans affairs cooperative study group on antihypertensive agents. *Am J Hypertens*. 1996;9:187S-191S
59. Wright JT, Jr., Bakris G, Greene T, Agodoa LY, Appel LJ, Charleston J, Cheek D, Douglas-Baltimore JG, Gassman J, Glassock R, Hebert L, Jamerson K, Lewis J, Phillips RA, Toto RD, Middleton JP, Rostand SG. Effect of blood pressure lowering and antihypertensive drug class on progression of hypertensive kidney disease: Results from the aask trial. *JAMA*. 2002;288:2421-2431
60. Hansson L, Zanchetti A, Carruthers SG, Dahlof B, Elmfeldt D, Julius S, Menard J, Rahn KH, Wedel H, Westerling S. Effects of intensive blood-pressure lowering and low-dose aspirin in patients with hypertension: Principal results of the hypertension optimal treatment (hot) randomised trial. Hot study group. *Lancet*. 1998;351:1755-1762
61. Prevention of stroke by antihypertensive drug treatment in older persons with isolated systolic hypertension. Final results of the systolic hypertension in the elderly program (shep). Shep cooperative research group. *JAMA*. 1991;265:3255-3264
62. Luscher TF, Waeber B. Efficacy and safety of various combination therapies based on a calcium antagonist in essential hypertension: Results of a placebo-controlled randomized trial. *J Cardiovasc Pharmacol*. 1993;21:305-309
63. Sica DA. Rationale for fixed-dose combinations in the treatment of hypertension: The cycle repeats. *Drugs*. 2002;62:443-462
64. Frishman WH, Bryzinski BS, Coulson LR, DeQuattro VL, Vlachakis ND, Mroczek WJ, Dukart G, Goldberg JD, Alemayehu D, Koury K. A multifactorial trial design to assess combination therapy in hypertension. Treatment with bisoprolol and hydrochlorothiazide. *Arch Intern Med*. 1994;154:1461-1468
65. Pool JL, Cushman WC, Saini RK, Nwachuku CE, Battikha JP. Use of the factorial design and quadratic response surface models to evaluate the fosinopril and hydrochlorothiazide combination therapy in hypertension. *Am J Hypertens*. 1997;10:117-123
66. Roca-Cusachs A, Torres F, Horas M, Rios J, Calvo G, Delgadillo J, Teran M. Nitrendipine and enalapril combination therapy in mild to moderate hypertension: Assessment of dose-response relationship by a clinical trial of factorial design. *J Cardiovasc Pharmacol*. 2001;38:840-849
67. McGill JB, Reilly PA. Telmisartan plus hydrochlorothiazide versus telmisartan or hydrochlorothiazide monotherapy in patients with mild to moderate hypertension: A multicenter, randomized, double-blind, placebo-controlled, parallel-group trial. *Clin Ther*. 2001;23:833-850
68. Kochar M, Guthrie R, Triscari J, Kassler-Taub K, Reeves RA. Matrix study of irbesartan with hydrochlorothiazide in mild-to-moderate hypertension. *Am J Hypertens*. 1999;12:797-805
69. Nishizaka MK, Zaman MA, Calhoun DA. Efficacy of low-dose spironolactone in subjects with resistant hypertension. *Am J Hypertens*. 2003;16:925-930
70. Johnston SC, Sidney S, Hills NK, Grosvenor D, Klingman JG, Bernstein A, Levin E. Standardized discharge orders after stroke: Results of the quality improvement in stroke prevention (quisp) cluster randomized trial. *Ann Neurol*.67:579-589
71. Lynch JK, Hirtz DG, DeVeber G, Nelson KB. Report of the national institute of neurological disorders and stroke workshop on perinatal and childhood stroke. *Pediatrics*. 2002;109:116-123
72. Kreuter MW, Strecher VJ. Do tailored behavior change messages enhance the effectiveness of health risk appraisal? Results from a randomized trial. *Health Educ Res*. 1996;11:97-105

73. Kreuter MW, Strecher VJ, Glassman B. One size does not fit all: The case for tailoring print materials. *Ann Behav Med*. 1999;21:276-283
74. Lavori PW, Dawson R, Rush AJ. Flexible treatment strategies in chronic disease: Clinical and research implications. *Biol Psychiatry*. 2000;48:605-614
75. Haynes RB, McDonald H, Garg AX, Montague P. Interventions for helping patients to follow prescriptions for medications. *Cochrane Database Syst Rev*. 2002:CD000011
76. Bosworth HB, Olsen MK, Gentry P, Orr M, Dudley T, McCant F, Oddone EZ. Nurse administered telephone intervention for blood pressure control: A patient-tailored multifactorial intervention. *Patient Educ Couns*. 2005;57:5-14
77. Bosworth HB, Olsen MK, Grubber JM, Neary AM, Orr MM, Powers BJ, Adams MB, Svetkey LP, Reed SD, Li Y, Dolor RJ, Oddone EZ. Two self-management interventions to improve hypertension control: A randomized trial. *Ann Intern Med*. 2009;151:687-695
78. Bandura A. *Social foundations of thought and action: A social cognitive theory.* Englewood Cliffs, NJ: Prentice Hall; 1986.
79. Bandura A. *Social learning theory.* Englewood Cliffs, NJ: Prentice Hall; 1977.
80. Bandura A. *Self-efficacy: The exercise of control*. New York: W.H. Freeman; 1997.
81. Prochaska JO, DiClemente CC. Common processes of self-change in smoking, weight control and psychological distress. . In: Shiffman S WT, ed. *Coping and substance use*. New York: Academic Press; 1985:345-363.
82. AbuSabha R, Achterberg C. Review of self-efficacy and locus of control for nutrition- and health-related behavior. *J Am Diet Assoc*. 1997;97:1122-1132
83. McAuley E. The role of efficacy cognitions in the prediction of exercise behavior in middle-aged adults. *J Behav Med*. 1992;15:65-88
84. McAuley E, Courneya KS, Rudolph DL, Lox CL. Enhancing exercise adherence in middle-aged males and females. *Prev Med*. 1994;23:498-506
85. Perri MG, Nezu AM, McKelvey WF, Shermer RL, Renjilian DA, Viegener BJ. Relapse prevention training and problem-solving therapy in the long-term management of obesity. *J Consult Clin Psychol*. 2001;69:722-726
86. Wing RR, Jeffery RW. Benefits of recruiting participants with friends and increasing social support for weight loss and maintenance. *J Consult Clin Psychol*. 1999;67:132-138
87. Dishman RK, Sallis JF. Determinants and interventions for physical activity and exercise. In: Bouchard C SR, Stephens T., ed. *Physical activity, fitness, and health: International proceedings and consensus statement.* Champaign, IL: Human Kinetics Publishers; 1994:214-238.
88. Britt E, Hudson SM, Blampied NM. Motivational interviewing in health settings: A review. *Patient Educ Couns*. 2004;53:147-155
89. Burke BL, Arkowitz H, Menchola M. The efficacy of motivational interviewing: A meta-analysis of controlled clinical trials. *J Consult Clin Psychol*. 2003;71:843-861
90. Serdula M, Coates R, Byers T, Mokdad A, Jewell S, Chavez N, Mares-Perlman J, Newcomb P, Ritenbaugh C, Treiber F, et al. Evaluation of a brief telephone questionnaire to estimate fruit and vegetable consumption in diverse study populations. *Epidemiology*. 1993;4:455-463
91. Block G, Gillespie C, Rosenbaum EH, Jenson C. A rapid food screener to assess fat and fruit and vegetable intake. *Am J Prev Med*. 2000;18:284-288
92. Thygesen K, Alpert JS, White HD, Jaffe AS, Apple FS, Galvani M, Katus HA, Newby LK, Ravkilde J, Chaitman B, Clemmensen PM, Dellborg M, Hod H, Porela P, Underwood R, Bax JJ, Beller GA, Bonow R, Van der Wall EE, Bassand JP, Wijns W, Ferguson TB, Steg PG, Uretsky BF, Williams DO, Armstrong PW, Antman EM, Fox KA, Hamm CW, Ohman EM, Simoons ML, Poole-Wilson PA, Gurfinkel EP, Lopez-Sendon JL, Pais P, Mendis S, Zhu JR, Wallentin LC, Fernandez-Aviles F, Fox KM, Parkhomenko AN, Priori SG, Tendera M, Voipio-Pulkki LM, Vahanian A, Camm AJ, De Caterina R, Dean V, Dickstein K, Filippatos G, Funck-Brentano C, Hellemans I, Kristensen SD, McGregor K, Sechtem U, Silber S, Widimsky P, Zamorano JL, Morais J, Brener S, Harrington R, Morrow D, Lim M, Martinez-Rios MA, Steinhubl S, Levine GN, Gibler WB, Goff D, Tubaro M, Dudek D, Al-Attar N. Universal definition of myocardial infarction. *Circulation*. 2007;116:2634-2653

93. Hulley S, Grady D, Bush T, Furberg C, Herrington D, Riggs B, Vittinghoff E. Randomized trial of estrogen plus progestin for secondary prevention of coronary heart disease in postmenopausal women. Heart and estrogen/progestin replacement study (hers) research group. *JAMA*. 1998;280:605-613
94. Grady D, Applegate W, Bush T, Furberg C, Riggs B, Hulley SB. Heart and estrogen/progestin replacement study (hers): Design, methods, and baseline characteristics. *Control Clin Trials*. 1998;19:314-335
95. Yusuf S, Zhao F, Mehta SR, Chrolavicius S, Tognoni G, Fox KK. Effects of clopidogrel in addition to aspirin in patients with acute coronary syndromes without st-segment elevation. *N Engl J Med*. 2001;345:494-502
96. Miller WR, Yahne CE, Moyers TB, Martinez J, Pirritano M. A randomized trial of methods to help clinicians learn motivational interviewing. *J Consult Clin Psychol*. 2004;72:1050-1062
97. Moyers TB, Martin T, Manuel JK, Hendrickson SM, Miller WR. Assessing competence in the use of motivational interviewing. *J Subst Abuse Treat*. 2005;28:19-26
98. Heisler M, Hofer TP, Klamerus ML, Schmittdiel J, Selby J, Hogan MM, Bosworth HB, Tremblay A, Kerr EA. Study protocol: The adherence and intensification of medications (aim) study--a cluster randomized controlled effectiveness study. *Trials*. 2010;11:95
