# Supplementary material for: Effect of Lifestyle Coaching or Enhanced Pharmacotherapy on Blood Pressure Control Among Black Adults With Persistent Uncontrolled Hypertension: A Cluster Randomized Clinical Trial
Source: JAMA Netw Open. 2022 May 18;5(5):e2212397. doi: 10.1001/jamanetworkopen.2022.12397 (PMC9118047; doi:10.1001/jamanetworkopen.2022.12397)
Supplement: Supplement 2. — eMethods. Treatment Groups and Statistical Analysis eResults. Sensitivity Analysis eTable 1. Distribution of Mean Blood Pressure at Baseline and 12, 24, and 48 Months Post Enrollment by Study Group eTable 2. Timing of Blood Pressure Measurements Carried Forward at Each Follow-up Period eTable 3. Blood Pressure Outcomes at 12, 24, and 48 Months Post Enrollment, Including 81 Additional Subjects Who Declined to Participate in EP (n = 26) or LC (n = 55) Group but Allowed EMR Data Use eTable 4. Blood Pressure Outcomes at 12, 24, and 48 Months Post Enrollment Adjusted for Physician Patient Panel Size eTable 5. Blood Pressure Outcomes at 12, 24, and 48 Months Post Enrollment Among Participants Who Had Available Blood Pressure Data Recorded During the Study Period eTable 6. Models of Blood Pressure Control at 12, 24, and 48 Months Post Enrollment Stratified by Sex and Age eTable 7. Blood Pressure Outcomes at 12, 24, and 48 Months Post Enrollment With Alternate Cut Point for Blood Pressure Control (<130/80) eTable 8. Blood Pressure Outcomes at 12, 24, and 48 Months Post Enrollment With Models Using Multiple Imputations eTable 9. Sensitivity Analysis for Blood Pressure Outcomes at 48 Months Post Enrollment eTable 10. Outcomes Across the Groups for Outpatient Utilization of Primary Care Services, Clinician Prescribing Pattern for Antihypertensives, Participant Compliance With Refills, Body Mass Index, or Self-reported Physical Activity eTable 11. Types of Antihypertensive Prescribed Across the 3 Groups eTable 12. Clinical Outcomes During Postintervention Follow-up eFigure 1. Probabilities of Controlled BP Over Time for Usual Care, EP Group, and LC Group Based on the Generalized Linear Mixed-Effect Model eFigure 2. Adherence to Lifestyle Coaching Sessions (n = 286) [file jamanetwopen-e2212397-s002.pdf]

Supplementary Online Content

Nguyen-Huynh MN, Young JD, Ovbiagele B, et al. Effect of lifestyle coaching or enhanced pharmacotherapy on blood pressure control among Black adults with persistent uncontrolled hypertension: a cluster randomized clinical trial. *JAMA Netw Open*. 2022;5(5):e2212397. doi:10.1001/jamanetworkopen.2022.12397

eMethods. Treatment Groups and Statistical Analysis
eResults. Sensitivity Analysis
eTable 1. Distribution of Mean Blood Pressure at Baseline and 12, 24, and 48 Months Post Enrollment by Study Group
eTable 2. Timing of Blood Pressure Measurements Carried Forward at Each Follow-up Period
eTable 3. Blood Pressure Outcomes at 12, 24, and 48 Months Post Enrollment, Including 81 Additional Subjects Who Declined to Participate in EP (n = 26) or LC (n = 55) Group but Allowed EMR Data Use
eTable 4. Blood Pressure Outcomes at 12, 24, and 48 Months Post Enrollment Adjusted for Physician Patient Panel Size
eTable 5. Blood Pressure Outcomes at 12, 24, and 48 Months Post Enrollment Among Participants Who Had Available Blood Pressure Data Recorded During the Study Period
eTable 6. Models of Blood Pressure Control at 12, 24, and 48 Months Post Enrollment Stratified by Sex and Age
eTable 7. Blood Pressure Outcomes at 12, 24, and 48 Months Post Enrollment With Alternate Cut Point for Blood Pressure Control (<130/80)
eTable 8. Blood Pressure Outcomes at 12, 24, and 48 Months Post Enrollment With Models Using Multiple Imputations
eTable 9. Sensitivity Analysis for Blood Pressure Outcomes at 48 Months Post Enrollment
eTable 10. Outcomes Across the Groups for Outpatient Utilization of Primary Care Services, Clinician Prescribing Pattern for Antihypertensives, Participant Compliance With Refills, Body Mass Index, or Self-reported Physical Activity
eTable 11. Types of Antihypertensive Prescribed Across the 3 Groups
eTable 12. Clinical Outcomes During Postintervention Follow-up
eFigure 1. Probabilities of Controlled BP Over Time for Usual Care, EP Group, and LC Group Based on the Generalized Linear Mixed-Effect Model
eFigure 2. Adherence to LC Sessions (n = 286)

This supplementary material has been provided by the authors to give readers additional information about their work.

© 2022 Nguyen-Huynh MN et al. *JAMA Network Open*.

eMethods. Treatment Groups and Statistical Analysis

Intervention arms.

Enhanced arm: Weekly lists of adults with uncontrolled BP were generated and used by the research nurse coordinator to outreach and assisted with appointment scheduling for blood pressure (BP) check visits. A pharmacist reviewed patient progress and BP medication regimen at each visit, with adjustments made as needed. Those who remained uncontrolled were scheduled to return every 2-3 weeks until BP was under control. Every attempt was also made to reschedule promptly whenever a participant could not make an appointment. Education materials made available to the participants included the guidebook on "Caring for Your Family's Health: A Guide for African Americans" and DVD, Kaiser Permanente Northern California (KPNC) handout on hypertension (HTN) and the importance of controlling BP.

Lifestyle arm: All enrolled participants could receive up to 16 phone-based coaching sessions. With every session, the coaches worked on developing rapport with the participant and establishing an environment of collaboration, mutual respect, and trust. The initial 8 coaching sessions focused on topics such as the DASH eating plan, tracking food and food labels, portion sizes, healthy dining, physical activity, managing stress, turning setbacks into success, and staying motivated. Every session also included follow-up on prior goals, discussion of barriers to success, setting goals for the following week, and culturally appropriate recipes. The remaining 8 sessions allowed participants to work on any of the prior topics. In addition, education materials made available to the participants included the guidebook on "Caring for Your Family's Health: A Guide for African Americans" and DVD, KPNC handouts on DASH eating plan, and the study workbook.

Study staff training: Prior to starting the trial, all study staff were trained on stroke knowledge including warning signs and symptoms and risk factors, secondary stroke prevention measures specific for HTN, and MI training by a master trainer from KPNC Regional Health Education department. Research nurse coordinator and lifestyle coaches received individual phone supervision and/or additional booster sessions every 3 months by the master trainer. The master trainer listened to a random number of recorded lifestyle coaching sessions every 3 months and provided feedback to the coaches. However, the exact information regarding the feedback was not captured systematically. These feedback sessions also provided a forum for problem solving barriers to effective counseling, role-playing, content reinforcement and deepening understanding of MI.

Statistical analysis.

Multiple imputation models included age, sex, marital status, household income, history of chronic conditions (coronary artery disease, diabetes, stroke, heart failure), body mass index (BMI), smoking status, diastolic and systolic BP at 6, 12, 18, 24, 30, 36, 42, 48 months. BP control was then categorized as yes/no based on the values diastolic BP and systolic BP at 12, 24, and 48 months in each imputed dataset. The cutoff for statistical significance was 0.05.

© 2022 Nguyen-Huynh MN et al. *JAMA Network Open*.

eResults. Sensitivity Analysis

In a sensitivity analysis that based the outcome of BP control on estimated average yearly BP using the AUC method (Table 3A), the proportion of participants with controlled BP was 51.6% for UC, 52.0% for EP, and 60.5% for LC (EP vs. UC: *P*=0.95, LC vs. UC: *P*=0.012, EP vs LC: *P*=0.045). In another sensitivity analysis where BP control was considered as a longitudinal outcome, there was no significant difference in data on outcome prevalence at enrollment across the study arms were inconclusive (Wald-based *P*=0.05), but significantly different time trends for the 3 arms over time (Wald-based test of interaction *P*=0.03). Using the estimates from the main effect for the time trend and interaction term between time and trial arm, estimated odds ratios for the time trend for each of the 3 arms were UC=1.02 (95% CI, 1.00-1.05); EP=0.97 (95% CI, 0.93-1.02); LC=1.08 (95% CI, 1.02-1.14), indicating that increasing time was significantly associated with increased proportion with BP control for LC and slightly so for UC and a slight decrease in BP control for EP (eTable 9B).

© 2022 Nguyen-Huynh MN et al. *JAMA Network Open*.

**eTable 1. Distribution of Mean Blood Pressure at Baseline and 12, 24, and 48 Months Post Enrollment by Study Group**				
			**Lower 95%**	**Upper 95%**
	**N**	**Mean**	**CL for Mean**	**CL for Mean**
**Baseline**				
**Systolic**				
Usual care	1129	150.49	149.80	151.18
Enhanced	316	151.47	150.24	152.70
Lifestyle	236	149.50	148.23	150.77
**Diastolic**				
Usual care	1129	84.53	83.84	85.22
Enhanced	316	84.65	83.33	85.96
Lifestyle	236	84.40	82.95	85.84
**12 months post-enrollment**				
**Systolic**				
Usual care	1057	135.7	134.8	136.6
Enhanced	328	135.0	133.4	136.6
Lifestyle	273	134.2	132.4	136.0
**Diastolic**				
Usual care	1057	76.36	75.67	77.05
Enhanced	328	75.23	73.98	76.49
Lifestyle	273	73.68	72.21	75.14
**24 months post-enrollment**				
**Systolic**				
Usual care	1090	136.0	135.1	136.9
Enhanced	336	135.4	133.8	137.0
Lifestyle	280	133.3	131.5	135.0
**Diastolic**				
Usual care	1090	75.62	74.93	76.30
Enhanced	336	75.01	73.79	76.24
Lifestyle	280	73.98	72.70	75.27
**48 months post-enrollment**				
**Systolic**				
Usual care	1101	135.1	134.2	136.1
Enhanced	340	135.1	133.4	136.8
Lifestyle	283	132.7	130.8	134.6
**Diastolic**				
Usual care	1101	74.49	74.49	75.22
Enhanced	340	73.41	73.41	74.68

© 2022 Nguyen-Huynh MN et al. *JAMA Network Open*.

Lifestyle	283	73.30	73.30	74.71

eTable 2. Timing of Blood Pressure Measurements Carried Forward at EACH FOLLOW-up Period

	Usual Care (n = 1,129)	Enhanced Monitoring (n = 346)	Lifestyle Coaching (n = 286)
**12-month post-enrollment follow-up**			
Number of participants with BP carried forward	230 (20.4%)	79 (22.8%)	56 (19.6%)
Timing of BP measurement used, months since enrollment, mean (±SD)	5.18 (2.69)	6.09 (2.68)	5.61 (2.55)
**24-month post-enrollment follow-up**			
Number of participants with BP carried forward	323 (28.6%)	99 (28.6%)	83 (29.0%)
Timing of BP measurement used, months since enrollment, mean (±SD)	13.87 (5.95)	15.49 (5.13)	14.86 (6.10)
**48-month post-enrollment follow-up**			
Number of participants with BP carried forward	396 (35.1%)	117 (33.8%)	82 (28.7%)
Timing of BP measurement used, months since enrollment, mean (±SD)	30.27 (13.41)	32.0 (12.52)	31.29 (13.57)

© 2022 Nguyen-Huynh MN et al. *JAMA Network Open*.

eTable 3. Blood Pressure Outcomes at 12, 24, and 48 Months Post Enrollment, Including 81 Additional Subjects Who Declined to Participate in EP (n = 26) or LC (n = 55) Group but Allowed EMR Data Use

	Odds Ratio	95% C.I.		*P* value
**Blood pressure outcomes at 12 months post-enrollment**				
Usual Care	reference	-	-	-
Enhanced	1.08	0.85	1.38	0.52
Lifestyle	1.41	1.09	1.83	0.01
**Blood pressure outcomes at 24 months post-enrollment**				
Usual Care	reference	-	-	-
Enhanced	1.25	0.98	1.6	0.07
Lifestyle	1.58	1.21	2.06	<0.001
**Blood pressure outcomes at 48 months post-enrollment**				
Usual Care	reference	-	-	-
Enhanced	1.07	0.83	1.39	0.60
Lifestyle	1.45	1.08	1.94	0.01

© 2022 Nguyen-Huynh MN et al. *JAMA Network Open*.

eTable 4. Blood Pressure Outcomes at 12, 24, and 48 Months Post Enrollment Adjusted for Physician Patient Panel Size

	Usual Care (n = 1,129)	Enhanced Monitoring (n = 346)	Lifestyle Coaching (n = 286)	*P* value		
				Enhanced vs. Usual Care	Lifestyle vs. Usual Care	Enhanced vs. Lifestyle
**Blood pressure outcomes at 12-month post-enrollment follow-up**						
Number of participants with BP control	698	223	194		-	-
Percent of participants with BP control (of participants enrolled)	54.1%	56.2%	60.4%	0.44	0.07	0.37
	[38.7, 68.7]	[41.0, 71.1]	[44.0, 74.7]			
Adjusted odds ratio (95% confidence interval)	Reference	1.11	1.3	-	-	-
		[0.85, 1.44]	[0.98, 1.72]			
**Blood pressure outcomes at 24-month post-enrollment follow-up ***						
Number of participants with BP control	691	234	207	-	-	-
Percent of participants with BP Control (of participants enrolled)	60.3	66.4	72.2	0.09	0.001	0.17
	[55.9, 64.6]	[60.0, 72.3]	[65.7, 77.9]			
Adjusted odds ratio (95% confidence interval)	Reference	1.3	1.71	-	-	-
		[0.96, 1.76]	[1.23, 2.37]			
**Blood pressure outcomes at 48-month post-enrollment follow-up**						
Number of participants with BP control	728	230	209	-	-	-
Percent of participants with BP Control (of participants enrolled)	64.4	66.1%	72,9%	0.562	0.007	0.07
	[0.49, 0.77]	[51.0, 78.5]	[58.2, 83.9]			
Adjusted odds ratio (95% confidence interval)	Reference	1.08	1.49	-	-	-
		[0.84, 1.39]	[1.12, 1.99]			

Notes: For blood pressure outcomes at: the end of the12-month intervention, we selected one BP reading between 0-15 months, but closest to 12 months post-enrollment; 24-month post-enrollment follow-up, we selected one BP reading that was between 0-27 months, but closest to 24 months post-enrollment; and 48-month post-enrollment follow-up, we selected one BP reading that was between 0-51 months, but closest to 48 months post-enrollment. Provider patient panel size was categorized as 1-10, 11-50, 50-100, 101+; 95% confidence intervals and odds ratios account for clustering by provider using generalized linear mixed models. BP, blood pressure.

* Excluded n=3 participants where provider panel size was <11

© 2022 Nguyen-Huynh MN et al. *JAMA Network Open*.

eTable 5. Blood Pressure Outcomes at 12, 24, and 48 Months Post Enrollment Among Participants Who Had Available Blood Pressure Data Recorded During the Study Period

	Usual Care	Enhanced Monitoring	Lifestyle Coaching	*P* value		
				Enhanced vs. Usual Care	Lifestyle vs. Usual Care	Enhanced vs. Lifestyle
**Blood pressure outcomes at 12 months post-enrollment**						
Number who had BP reading within 15 months	1057	328	273	-	-	-
Number of participants with BP control	698	223	194	-	-	-
Percent of participants with BP control (of participants with BP reading)	66.0% [63.0, 69.2]	68.0% [62.3, 72.9]	71.1% [65.1, 76.5]	0.59	0.14	0.41
**Blood pressure outcomes at 24 months post-enrollment**						
Number who had BP reading within 27 months	1090	336	280	-	-	-
Number of participants with BP control	691	234	207	-	-	-
Percent of participants with BP control (of participants with BP reading)	63.4% [60.5, 66.0]	69.6% [63.1, 75.5]	73.9% [66.9, 80.2]	0.08	0.007	0.34
**Blood pressure outcomes at 48 months post-enrollment**						
Number who had BP reading within 51 months	1101	340	283	-	-	-
Number of participants with BP control	728	230	209	-	-	-
Percent of participants with BP control (of participants with BP reading)	66.1% [63.4, 68.5]	67.6% [63.6, 71.7]	73.9% [69.2, 78.2]	0.47	0.005	0.05

Notes: For blood pressure outcomes at: the end of the12-month intervention, we selected one BP reading between 0-15 months, but closest to 12 months post-enrollment; 24-month post-enrollment follow-up, we selected one BP reading that was between 0-27 months, but closest to 24 months post-enrollment; and 48-month post-enrollment follow-up, we selected one BP reading that was between 0-51 months, but closest to 48 months post-enrollment. 95% confidence intervals account for clustering by provider using generalized linear mixed effect models. BP, blood pressure.

© 2022 Nguyen-Huynh MN et al. *JAMA Network Open*.

**eTable 6. Models of Blood Pressure Control at 12, 24, and 48 Months Post Enrollment Stratified by Sex and Age**		
	**Odds Ratio [95% C.I.]**	
	**Enhanced vs Usual Care**	**Lifestyle vs Usual Care**
**12 months post-enrollment**		
Female	1.05 [0.78, 1.41]	1.34 [0.95, 1.89]
Male	1.21 [0.70, 2.07]	1.25 [0.72, 2.16]
Less than 65 years	1.09 [0.77, 1.55]	1.22 [0.84, 1.79]
65 years and above	1.14 [0.75, 1.71]	1.4 [0.89, 2.20]
**24 months post-enrollment**		
Female	1.20 [0.89, 1.61]	1.62 [1.14, 2.29]
Male	1.77 [1.03, 3.03]	1.73 [1.01, 2.97]
Less than 65 years	1.33 [0.96, 1.85]	1.75 [1.20, 2.55]
65 years and above	1.31 [0.88, 1.97]	1.54 [0.99, 2.40]
**48 months post-enrollment**		
Female	1.06 [0.78, 1.45]	1.76 [1.22, 2.56]
Male	1.17 [0.72, 1.91]	1.08 [.67, 1.75]
Less than 65 years	1.03 [0.74, 1.42]	1.38 [0.95, 2.01]
65 years and above	1.20 [0.8, 1.81]	1.67 [1.06, 2.64]

© 2022 Nguyen-Huynh MN et al. *JAMA Network Open*.

eTable 7. Blood Pressure Outcomes at 12, 24, and 48 Months Post Enrollment With Alternate Cut Point for Blood Pressure Control (<130/80)

	Usual Care (n = 1,129)	Enhanced Monitoring (n = 346)	Lifestyle Coaching (n = 286)	*P* value		
				Enhanced vs. Usual Care	Lifestyle vs. Usual Care	Enhanced vs. Lifestyle
**Blood pressure outcomes at 12 months post-enrollment**						
Number of participants with BP control	271	91	73	-	-	-
Percent of participants with BP control (of participants enrolled)	24.10% [21.6%, 26.7%]	26.40% [21.9%, 31.4%]	25.50% [20.8%, 31.0%]	0.39	0.61	0.81
Adjusted odds ratio (95% confidence interval)	Reference	1.13 [0.85, 1.50]	1.08 [0.8, 1.47]	-	-	-
**Blood pressure outcomes at 24 months post-enrollment**						
Number of participants with BP control	277	86	85	-	-	-
Percent of participants with BP Control (of participants enrolled)	24.50% [22.1%, 27.1%]	24.90% [20.6%, 29.7%]	29.70% [24.7%, 35.3]	0.90	0.07	0.17
Adjusted odds ratio (95% confidence interval)	Reference	1.02 [0.77, 1.35]	1.3 [0.98, 1.73]	-	-	-
**Blood pressure outcomes at 48 months post-enrollment**						
Number of participants with BP control	289	97	88	-	-	-
Percent of participants with BP Control (of participants enrolled)	25.60% [22.9%, 28.5%]	28.30% [23.5%, 33.5%]	30.90% [25.6%, 36.7%]	0.36	0.09	0.49
Adjusted odds ratio (95% confidence interval)	Reference	1.15 [0.86, 1.53]	1.3 [0.96, 1.76]			

Notes: For blood pressure outcomes at: the end of the 12-month intervention, we selected one BP reading between 0-15 months, but closest to 12 months post-enrollment; 24-month post-enrollment follow-up, we selected one BP reading that was between 0-27 months, but closest to 24 months post-enrollment; and 48-month post-enrollment follow-up, we selected one BP reading that was between 0-51 months, but closest to 48 months post-enrollment; 95% confidence intervals and odds ratios account for clustering by provider using generalized linear mixed models. BP, blood pressure.

© 2022 Nguyen-Huynh MN et al. *JAMA Network Open*.

eTable 8. Blood Pressure Outcomes at 12, 24, and 48 Months Post Enrollment With Models Using Multiple Imputations

	Odds Ratio	95% C.I.		*P* value
**Blood pressure outcomes at 12-month post-enrollment**				
Usual Care	reference	-	-	-
Enhanced	1.11	0.83	1.62	0.48
Lifestyle	1.22	1.14	1.26	0.23
**Blood pressure outcomes at 24-month post-enrollment**				
Usual Care	reference	-	-	-
Enhanced	1.23	0.90	1.66	0.19
Lifestyle	1.46	1.06	2.02	0.02
**Blood pressure outcomes at 48-month post-enrollment**				
Usual Care	reference	-	-	-
Enhanced	0.97	0.71	1.32	0.84
Lifestyle	1.40	1.02	1.92	0.04

Notes: Imputation models included age, sex, history of chronic disease (diabetes, stroke, coronary artery disease, heart failure), household income,marital status, systolic and diastolic blood pressure (continuous variable) at 6, 12, 18, 24, 30, 36, 42, 48 months post-enrollment. Data was imputed separately for each study arm. Imputed datasets were used to categorize and model BP control as a dichotomous outcome at 12,24, and 48 months post-enrollment (Glimmix models).

© 2022 Nguyen-Huynh MN et al. *JAMA Network Open*.

eTable 9. Sensitivity Analysis For Blood Pressure Outcomes At 48 Months Post Enrollment

9A. Blood pressure outcomes at 48-month post-enrollment follow-up using area under the curve analysis

	Usual Care	Enhanced Monitoring	Lifestyle Coaching	*P* value: Enhanced vs. Usual Care	*P* value: Lifestyle vs. Usual Care	*P* value: Enhanced vs. Lifestyle
Number of participants enrolled	1129	346	286	-	-	-
Number who had BP reading within 51 months	1083	340	278	-	-	-
Number of participants with BP control	583	180	173	-	-	-
Percent with BP control (of participants enrolled)	51.6% [48.5, 55.0]	52.0% [46.1, 57.9]	60.5% [54.6, 66.5]	0.95	0.01	0.05
Percent with BP control (of participants with BP reading)	53.8% [50.8 57.1]	52.9% [47.1, 58.7]	62.2% [56.2, 68.1]	0.76	0.02	0.03

9B. Estimated odds ratios for blood pressure control from a longitudinal analysis of blood pressure outcomes after enrollment.

	Adjusted Odds Ratio	95% CI
Outcome level at time of intervention		
Usual Care (Reference group)	1.00	-
Enhanced	1.15	0.95, 1.40
Lifestyle	1.27	1.03, 1.56
Time trend over the study period		
Usual Care	1.02	1.00, 1.05
Enhanced	0.97	0.93, 1.02
Lifestyle	1.08	1.02, 1.14

Abbreviations: BP, blood pressure. Notes: BP control was defined as <140/90 mmHg, meeting both systolic and diastolic criteria. Calculated area under the curve for BP readings between enrollment and 51 months post-enrollment. n=60 did not have 2 or BP measurements during period. We fit a generalized linear mixed model with a logit link function to longitudinally collected blood pressure control outcomes using random effects to account for clustering by individual and provider. Main effects for trial arm, years since randomization (time trend) and interaction terms between trial arm and time were included in the models. A test of linear hypotheses for the interaction between trial arm and time (years since enrollment) indicates differences in the slopes across the 3 groups (interaction p-value = 0.026). For ease of interpretation, estimates from the overall time trend and interaction effects were combined so that time trends (slopes) for each trial arm are presented. Model-based estimates of the outcome probabilities (predicted population margins) and corresponding 95% confidence intervals for the 3 arms are: 53.1% [51.2,55.0] for the Usual Care; 54.1% [50.7,57.4] for the Enhanced arm; and 61.3% [57.8,64.8] for the Lifestyle arm. P-values correspond to a Wald-based F-test with denominator degrees of freedom estimated using the Containment method

© 2022 Nguyen-Huynh MN et al. *JAMA Network Open*.

eTable 10. Outcomes Across the Groups for Outpatient Utilization of Primary Care Services, Clinician Prescribing Pattern for Antihypertensives, Participant Compliance With Refills, Body Mass Index, or Self-reported Physical Activity

	Usual Care (N=1,129)	Enhanced Monitoring (N=346)	Lifestyle Coaching (N=286)	*P* value		
				Enhanced vs. Usual Care	Lifestyle vs. Usual Care	Enhanced vs. Lifestyle
**Average number of outpatient primary care visits per year between 12- and 48-months post-enrollment**						
Mean [95% CI]	2.11 [1.78, 2.50]	2.05 [1.70, 2.48]	1.98 [1.62, 2.42]	0.84	0.65	0.80
**Change in body mass index from 12-month to 24-month post-enrollment follow-up**						
Mean [95% CI]	-0.22 [-0.37, -0.07]	-0.19 [-0.43, 0.05]	-0.29 [-0.56, -0.4]	0.84	0.60	0.54
**Change in body mass index from 12-month to 48-month post-enrollment follow-up**						
Mean [95% CI]	-0.38 [-0.6, -015]	-0.21 [-0.57, 0.16]	-0.64 [-1.04, -0.24]	0.44	0.27	0.12
**Change in physical activity (minutes per week) from baseline to 12-month post-enrollment follow-up**						
Mean [95% CI]	9.58 [-4.7, 23.9]	12.0 [-15.6, 39.5]	9.3 [-18.5, 37.1]	0.88	0.99	0.89
**Change in physical activity (minutes per week) from baseline to 48-month post-enrollment follow-up**						
Mean [95% CI]	-1.18 [-17.8, 15.5]	-8.76 [-39.7, 22.2]	-27.44 -60.9, 6.0]	0.67	0.17	0.42
**Percent BP control at end of 12-month intervention, by the number of anti-hypertensives prescribed at baseline**						
< 2 drugs	57.2	60.6	66.7	0.56	0.19	-
≥ 2 drugs	68.3	68.1	69.0			
**Percent BP control at 24-month post-enrollment follow-up, by the number of anti-hypertensives prescribed at baseline**						
< 2 drugs	54.4	66.3	65.7	0.42	0.20	-
≥ 2 drugs	65.6	69.2	80.0			
**Percent compliance to anti-hypertensive refills between 12-month and 48-month post-enrollment**						
				***P* value**		
Mean [95% CI]	70.1 [67.4-72.7]	69.1 [64.2-74.0]	71.7 [66.4-76.9]	0.77		

© 2022 Nguyen-Huynh MN et al. *JAMA Network Open*.

eTable 11. Types of Antihypertensive Prescribed Across the 3 Groups				
	Usual Care	Enhanced Monitoring	Lifestyle Coaching	*P* value
**At baseline**				
Average number of anti-hypertensive (±SD)	26.4 (22.09)	27.1 (22.70)	26.7 (21.66)	0.84
Types of anti-hypertensive				
Ace-inhibitor	3.6 (5.52)	3.7 (5.84)	3.8 (5.35)	0.70
Angiostensin receptor blocker	2.5 (5.20)	2.7 (5.31)	2.6 (5.04)	0.45
Beta-blocker	4.0 (6.23)	4.5 (6.42)	4.2 (5.80)	0.13
Calcium channel blocker	4.5 (5.71)	4.5 (6.08)	4.0 (5.37)	0.58
Diuretic	5.0 (6.31)	4.9 (6.14)	5.2 (6.42)	0.91
**At 12 months post-enrollment**				
Average number of anti-hypertensive (±SD)	7.3 (6.39)	7.3 (6.31)	7.2 (6.07)	0.95
Types of anti-hypertensive				
Ace-inhibitor	1.0 (1.56)	1.0 (1.62)	1.0 (1.49)	0.78
Angiostensin receptor blocker	0.6 (1.39)	0.7 (1.38)	0.7 (1.35)	0.38
Beta-blocker	1.1 (1.88)	1.2 (1.88)	1.1 (1.68)	0.26
Calcium channel blocker	1.3 (1.75)	1.4 (1.92)	1.2 (1.61)	0.77
Diuretic	1.3 (1.89)	1.3 (1.73)	1.4 (1.85)	0.86
**At 24 months post-enrollment**				
Average number of anti-hypertensive (±SD)	14.1 (11.96)	14.4 (12.19)	14.1 (11.70)	0.94
Types of anti-hypertensive				
Ace-inhibitor	1.9 (2.91)	1.9 (3.02)	2.0 (2.83)	0.91
Angiostensin receptor blocker	1.3 (2.70)	1.4 (2.75)	1.3 (2.59)	0.39
Beta-blocker	2.1 (3.41)	2.4 (3.59)	2.1 (3.16)	0.29
Calcium channel blocker	2.5 (3.24)	2.6 (3.59)	2.3 (3.10)	0.71
Diuretic	2.7 (3.49)	2.5 (3.33)	2.7 (3.50)	0.93
**At 48 months post-enrollment**				
On at least one anti-hypertensive				
Average number of anti-hypertensive (±SD)	26.4 (22.09)	27.1 (22.70)	26.7 (21.66)	0.84
Types of anti-hypertensive				
Ace-inhibitor	3.6 (5.52)	3.7 (5.84)	3.8 (5.35)	0.70
Angiostensin receptor blocker	2.5 (5.20)	2.7 (5.31)	2.6 (5.04)	0.45
Beta-blocker	4.0 (6.23)	4.5 (6.42)	4.2 (5.80)	0.13
Calcium channel blocker	4.5 (5.71)	4.5 (6.08)	4.0 (5.37)	0.58
Diuretic	5.0 (6.31)	4.9 (6.14)	5.2 (6.42)	0.91

Assessed the total number of dispensed outpatient prescriptions within 120 days prior to enrollment and from enrollment to 12, 24 and 48 months

© 2022 Nguyen-Huynh MN et al. *JAMA Network Open*.

eTable 12. Clinical Outcomes During Postintervention Follow-up

	Usual Care N=1129	Enhanced Monitoring N=346	Lifestyle Coaching N=286	*P* value		
				Enhanced vs. Usual Care	Lifestyle vs. Usual Care	Enhanced vs. Lifestyle
**Clinical outcomes during 24-month post-enrollment follow-up, percent [95% C.I.]**						
mortality all-causes [a]	4.9%	5.4%	5.2%	0.73	0.85	0.93
	[4.1, 5.9]	[3.2, 9.0]	[2.9, 9.1]			
ischemic stroke [a]	1.4%	2.0%	1.8%	0.46	0.63	0.80
	[0.90, 2.2]	0.87, 4.6]	[0.83, 3.7]			
hemorrhagic stroke [b]	0.3%	0.1%	0.9%	0.61	0.21	0.25
	[0.1, 0.9]	[0.0, 2.3]	[0.3, 3.0]			
**Clinical outcomes during 48-month post-enrollment follow-up, percent [95% C.I.]**						
mortality all-causes [a]	9.6%	9.3%	10.5%	0.89	0.65	0.63
	[78.7, 11.6]	[6.4, 13.3]	[7.6, 14.2]			
ischemic stroke [a]	2.7%	2.9%	4.5%	0.82	0.09	0.26
	[1.8, 3.8]	[1.5, 5.4]	[2.8, 7.4]			
hemorrhagic stroke [b]	0.8%	0.1%	0.9%	0.26	0.84	0.25
	[0.4, 1.5]	[0.0, 2.3]	[0.3, 3.0]			

Note: [a] based on generalized linear mixed effect models accounting for clustering by provider; [b] based on logistic model using Firth penalized likelihood for rare events. BP, blood pressure.

© 2022 Nguyen-Huynh MN et al. *JAMA Network Open*.

eFigure 1. Probabilities of Controlled BP Over Time for Usual Care, EP Group, and LC Group Based on the Generalized Linear Mixed-Effect Model

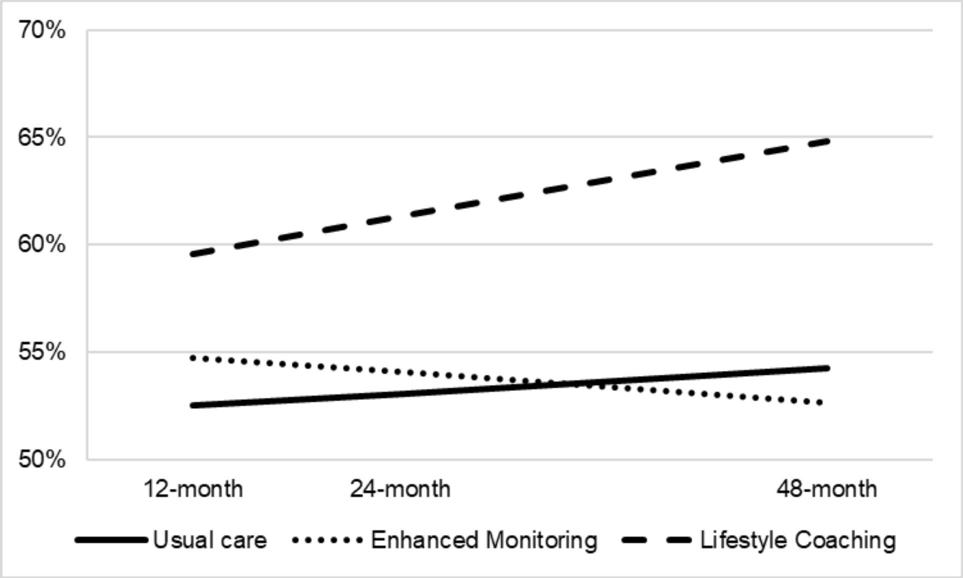

© 2022 Nguyen-Huynh MN et al. *JAMA Network Open*.

eFigure 2. Adherence to Lifestyle Coaching Sessions (n = 286)

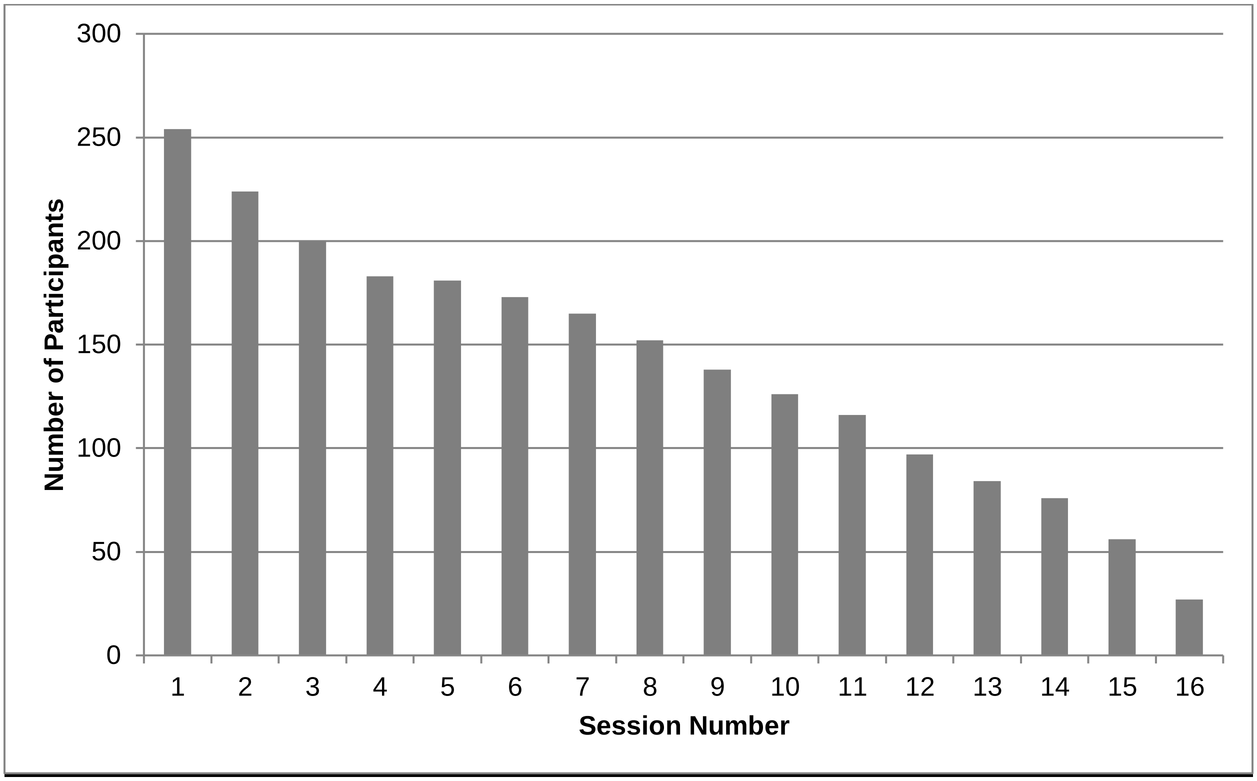

© 2022 Nguyen-Huynh MN et al. *JAMA Network Open*.
