# Supplementary material for: Effect of Lifestyle Coaching or Enhanced Pharmacotherapy on Blood Pressure Control Among Black Adults With Persistent Uncontrolled Hypertension: A Cluster Randomized Clinical Trial
Source: JAMA Netw Open. 2022 May 18;5(5):e2212397. doi: 10.1001/jamanetworkopen.2022.12397 (PMC9118047; doi:10.1001/jamanetworkopen.2022.12397)
Supplement: Supplement 3. — Data Sharing Statement [file jamanetwopen-e2212397-s00.pdf]

Data Sharing Statement

Nguyen-Huynh. Effect of Lifestyle Coaching or Enhanced Pharmacotherapy on Blood Pressure Control Among Black Adults With Persistent Uncontrolled Hypertension. *JAMA Netw Open*. Published May 18, 2022. doi:10.1001/jamanetworkopen.2022.12397

Data
Data available: No

Additional Information
Explanation for why data not available: if asked, may be considered on a case by case basis.
